# Supplementary material for: VASCilia is an open-source, deep learning-based tool for 3D analysis of cochlear hair cell stereocilia bundles
Source: PLoS Biol. 2026 Jan 20;24(1):e3003591. doi: 10.1371/journal.pbio.3003591 (PMC12829968; doi:10.1371/journal.pbio.3003591)
Supplement: S1 File — (PDF) [file pbio.3003591.s001.pdf]

Supplementary File 1

S0.1 VASCilia Workflow and Features

VASCilia begins by initializing all necessary properties for comprehensive analysis by invoking a function called initialize_ui, which sets up plugin buttons and prepares the user interface for operation.

Users can either open and preprocess a new dataset using the [Open Cochlea Datasets and Preprocess] button or upload an analyzed dataset with the [Upload Processed Stack] button. The plugin supports Zeiss (.czi), Leica (.lif), and Tagged Image (.tif) file formats, with the flexibility to incorporate additional formats. It starts by reading metadata and extracting physical resolution variables for later use in length computations, applies Contrast Limited Adaptive Histogram Equalization for pre-processing, and displays the channels as layers in Napari.

Users initiate their analysis by automatically trimming the stack to isolate the CCZ region of interest, aligning the stack with the tissue's planar polarity axis, and then proceeding with segmentation, reconstruction, and visualization. Subsequently, users can remove unwanted regions, perform measurements, calculate lengths from top to bottom of stereocilia bundles, compute fluorescence Intensity, predict the origin of the stack, determine orientation, and cluster rows into four categories (IHC, OHC1, OHC2, OHC3). All these functionalities depend on invoking a function called 'save_attributes,' which efficiently saves all variables used in the analysis in a pickle file. This storage action is implicit, requiring no manual save operations from the user, thus enabling the upload function to retrieve and apply these variables for visualization in the viewer. This setup enables analysts to resume or revisit their analysis at a later time.

After analyzing several datasets, users can compile all generated CSV files related to length computations, orientation, and fluorescence signal responses to analyze and plot observations necessary for their studies.

The user can reset Napari for analyzing a new dataset using the 'Reset' button. This feature saves all current variables and then clears them along with all existing layers. This process ensures that the plugin is thoroughly prepared and optimized to handle a new dataset to allow smooth transition between tasks.

S0.2 User-Enhanced Accuracy in Automated Measurements

In VASCilia, aside from the segmentation tasks, all automated measurements can be fine-tuned interactively by the user. The plugin is equipped with listeners that actively monitor user interactions related to the adjustment of points, that affect length and orientation computation. This feature ensures that any automated measurements can be further modified to reflect the precise requirements of the user.

Furthermore, for clustering task (cell type identification), VASCilia empowers users to intervene when automated clustering may not align perfectly with the expected outcomes. Users have the flexibility to reassign elements between clusters, correcting any discrepancies. This capability allows for significant refinement of the clustering results to ensure that the automated process is complemented by user expertise and judgment.

S0.3 Training section

VASCilia is fundamentally designed to obtain 3D segmentation of stereocilia bundles, an essential step for all subsequent measurements within the plugin. To ensure adaptability and utility across various labs, VASCilia includes a feature allowing users to fine-tune the existing segmentation model with additional images from their specific datasets. This adaptability is crucial for handling variations in staining techniques, settings, and image dimensions such as height, width, and resolution. This feature is both user-friendly and vital for broadening the plugin's applicability. The training module within VASCilia features seven buttons for ease of use:

Create/Save Ground Truth: allows users to generate a new layer, named 'Ground Truth', within the plugin. This button also enables saving of manually annotated data directly within this layer.

Copy Segmentation Masks to Ground Truth: simplifies the annotation process by transferring existing segmented 3D masks to the Ground Truth layer. This functionality allows users to make precise adjustments to the model's initial predictions rather than starting from scratch to simplify and save the time for the refinement process.

Generate Ground Truth Masks: initiates by identifying and correcting boundary-touching errors, zeroing out pixels where segmented labels overlap. This step is critical to ensure that each pixel retains a unique ID. Furthermore, the function includes a filtering mechanism to manage manually segmented masks sharing identical IDs, maintaining only the largest connected components to ensure each bundle has a distinct ID. This methodical approach is vital for preparing precise ground truth data necessary for effective training processes. Finally, all the masks are saved in a folder pre-defined in the configuration.

Display Stored Ground Masks: allows users to review the stored masks following the automated refinement. This step ensures that all bundles are correctly identified with IDs

Move Ground Truth to Training Folder: automates the transfer of all samples into a pre-configured folder designated for storing training data. This feature is designed to eliminate the need for manual copy-paste operations.

Check Training Data: performs a comprehensive verification of the training data. It ensures that each raw image is paired with a corresponding ground truth and confirms the existence of 'Train' and 'Val' folders within the configured directories, each containing distinct files. Additionally, this function uploads all masks to verify their uniqueness by checking for unique IDs. Should any issues be detected, the plugin will alert the user with a notification of the problem. Conversely, if all checks are passed successfully, the function will display a congratulatory prompt, asking the user to proceed with the training process.

Train New Model for 3DBundle Seg: initiates the training process for the segmentation algorithm. Users have the flexibility to train various models and select the most effective one by simply modifying a path in the configuration file. This plugin is open-source, enabling any user with Python expertise to extend its functionality to suit more specific research needs.
