# Supplementary figures and images for: VASCilia is an open-source, deep learning-based tool for 3D analysis of cochlear hair cell stereocilia bundles
Source: PLoS Biol. 2026 Jan 20;24(1):e3003591. doi: 10.1371/journal.pbio.3003591 (PMC12829968; doi:10.1371/journal.pbio.3003591)

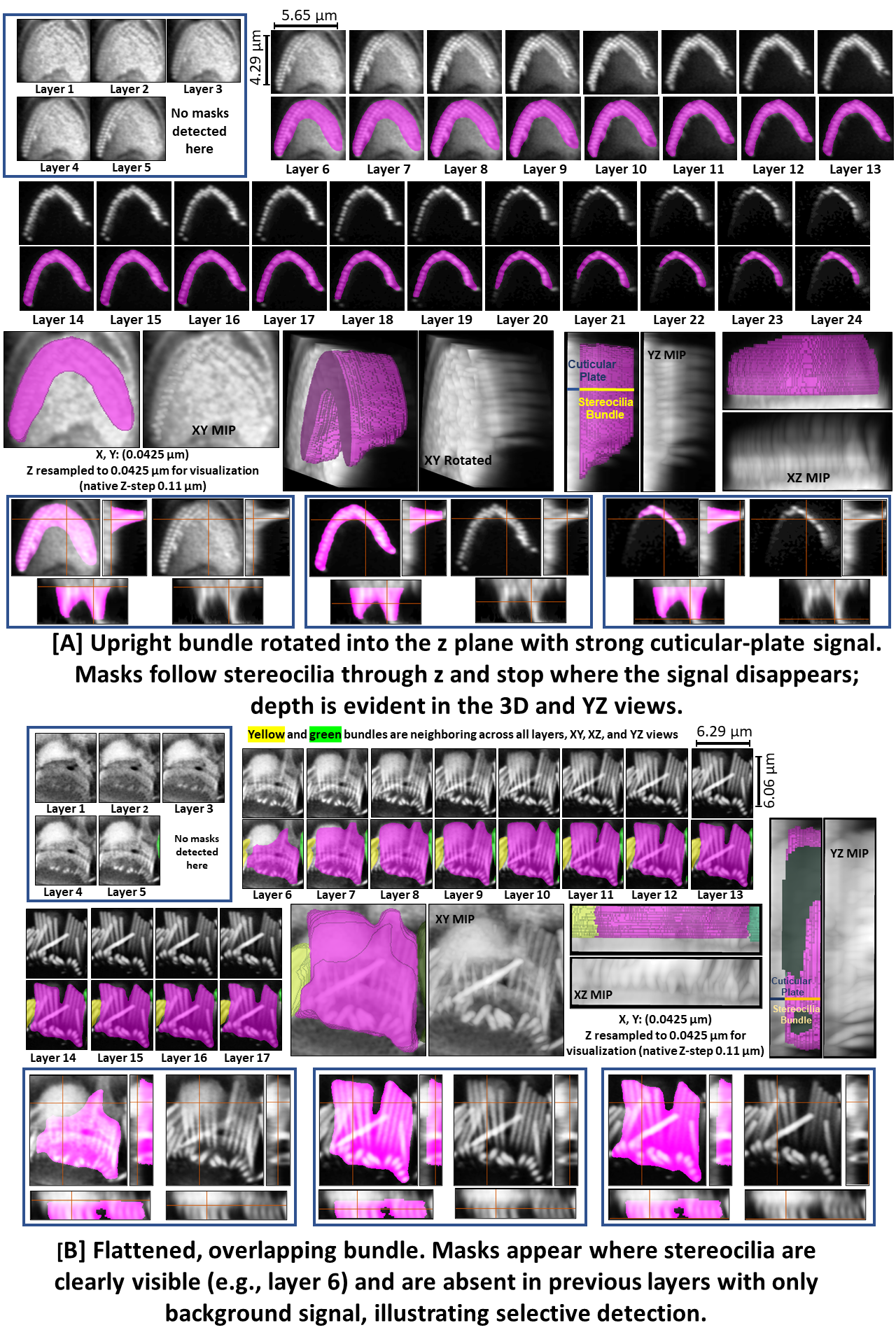

Supplement: S1 Fig — (TIFF) [file pbio.3003591.s002.tif]

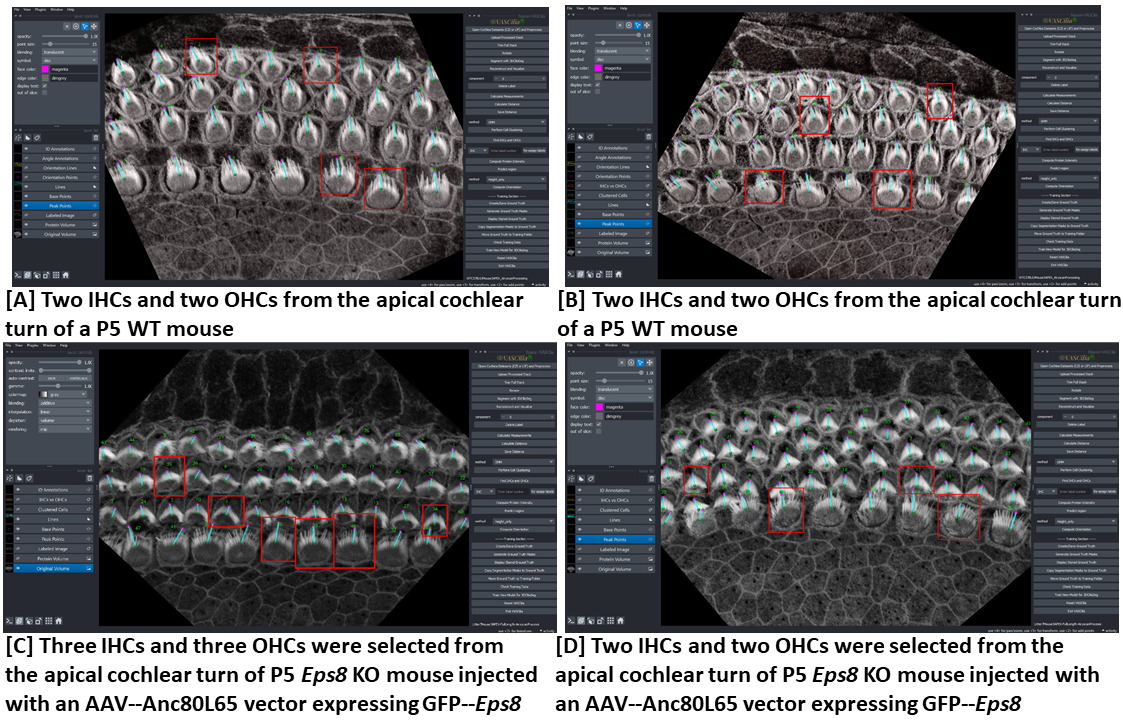

Supplement: S2 Fig — (TIFF) [file pbio.3003591.s003.tif]

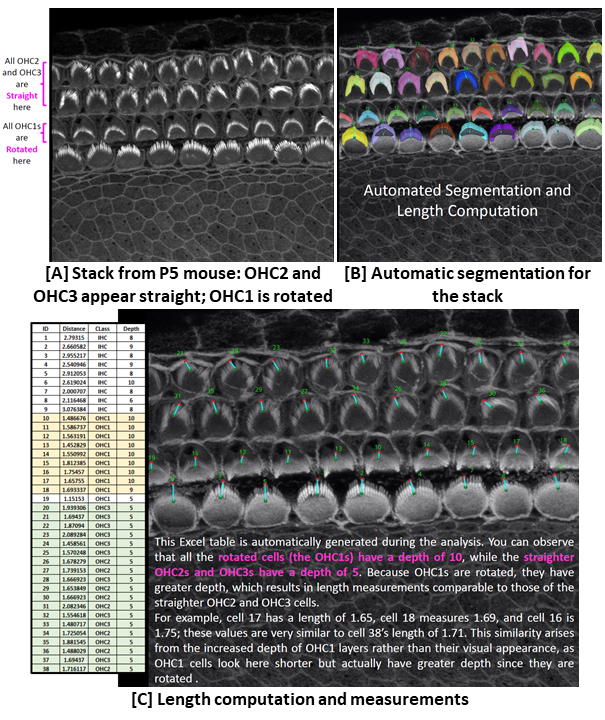

Supplement: S3 Fig — (TIFF) [file pbio.3003591.s004.tif]

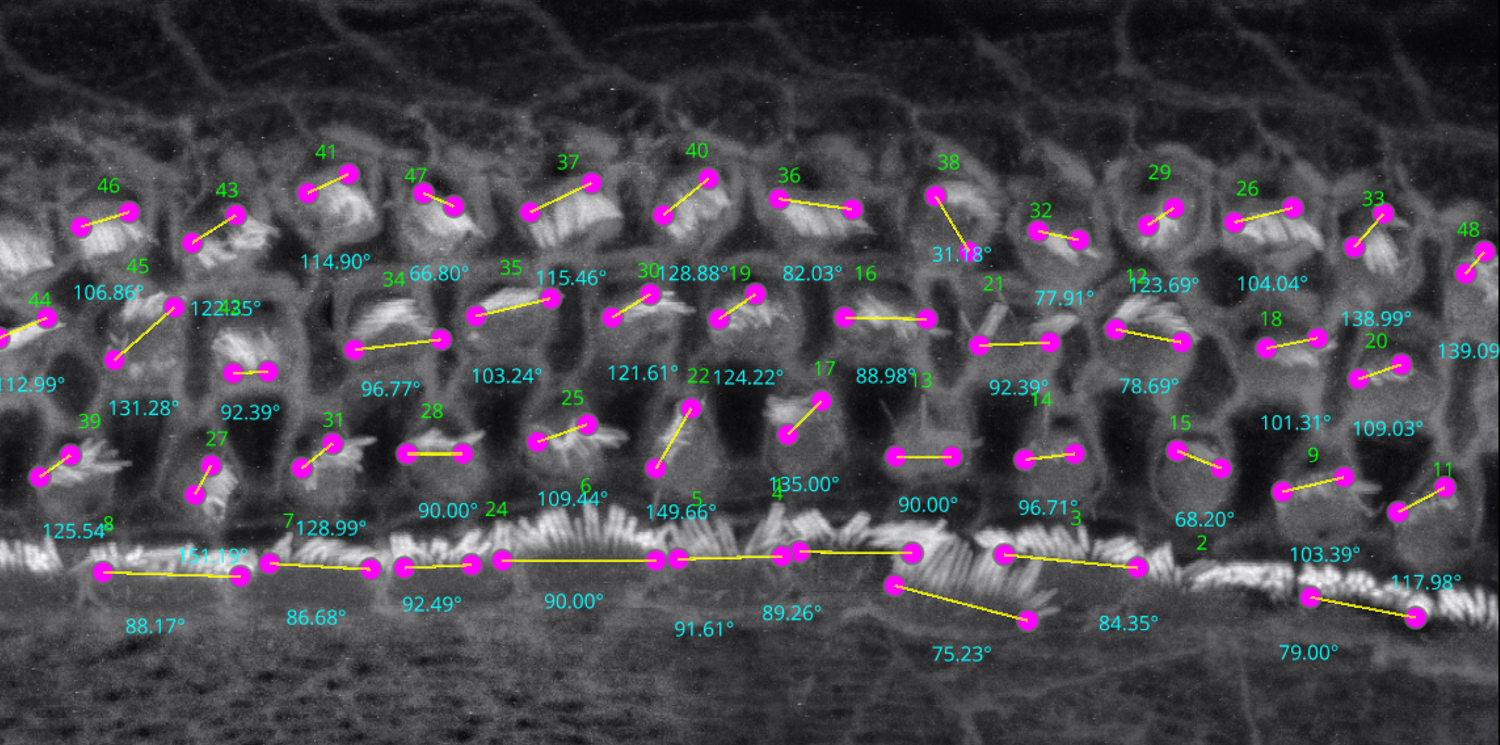

Supplement: S4 Fig — (TIFF) [file pbio.3003591.s005.tif]

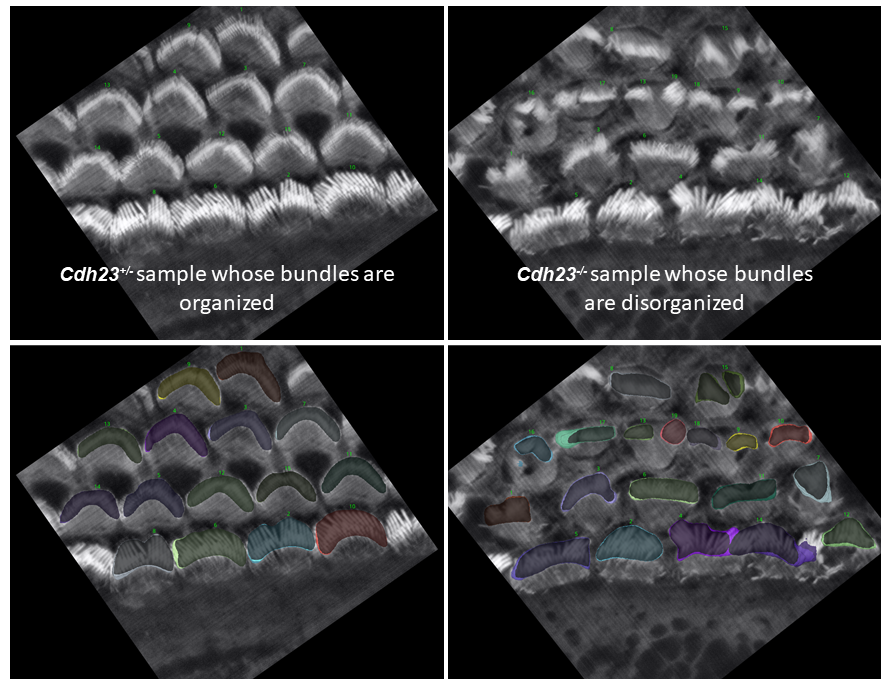

Supplement: S5 Fig — (TIFF) [file pbio.3003591.s006.tif]

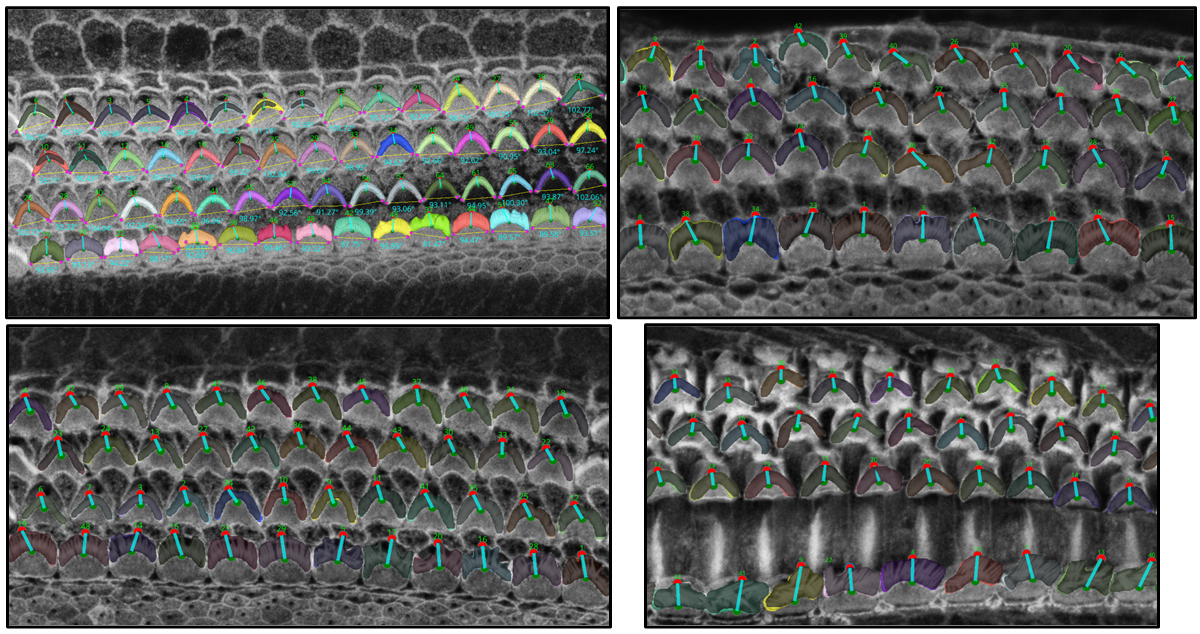

Supplement: S6 Fig — (TIFF) [file pbio.3003591.s007.tif]

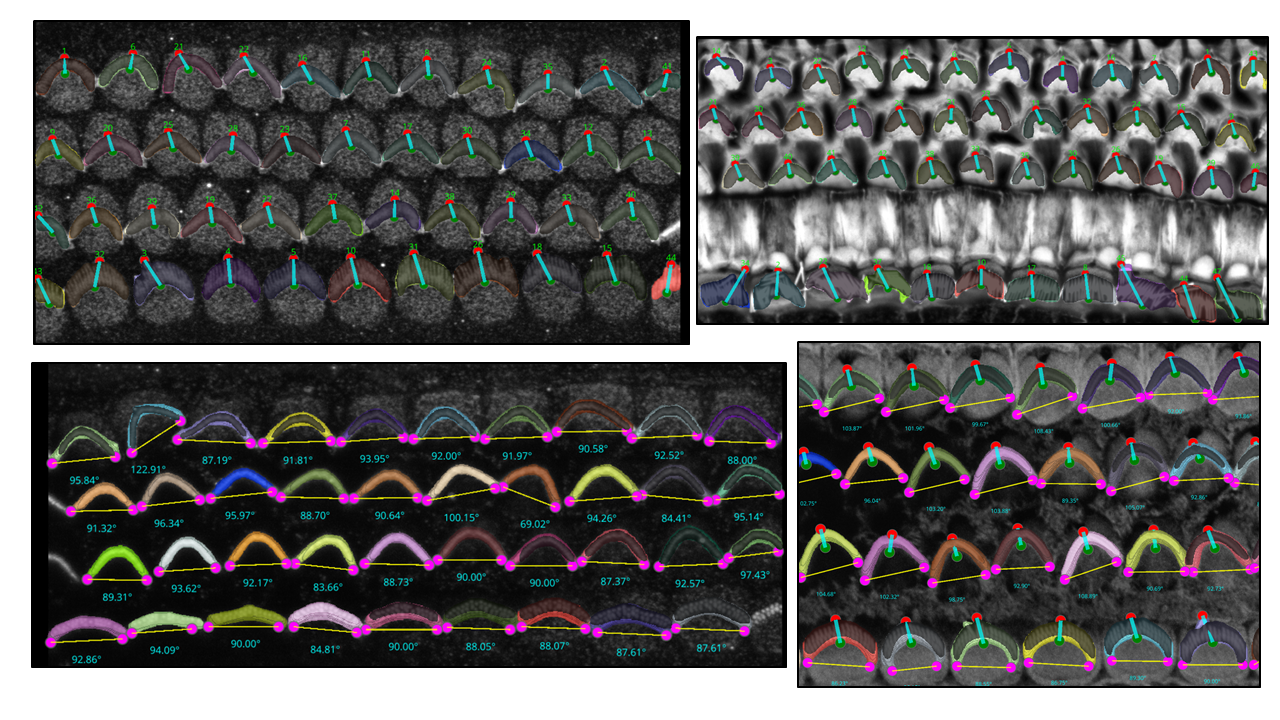

Supplement: S7 Fig — (TIFF) [file pbio.3003591.s008.tif]

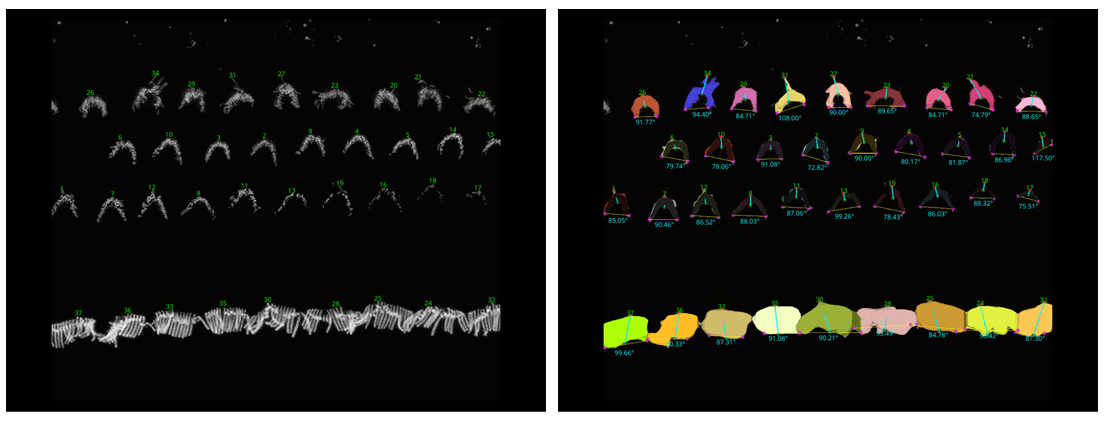

Supplement: S8 Fig — (TIFF) [file pbio.3003591.s009.tif]

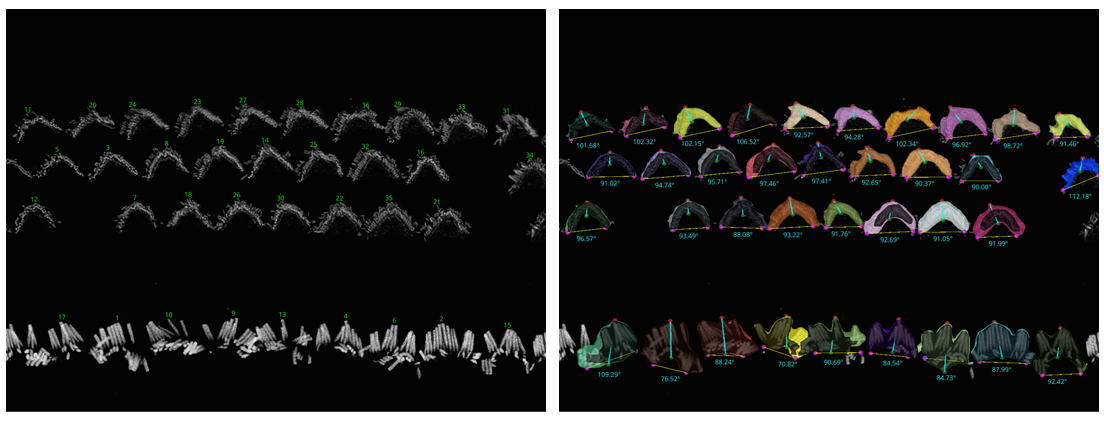

Supplement: S9 Fig — (TIFF) [file pbio.3003591.s010.tif]

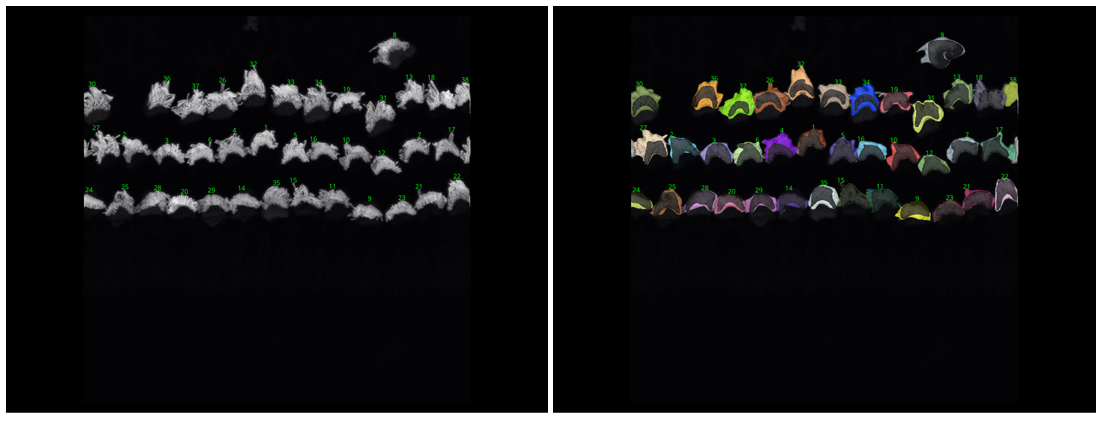

Supplement: S10 Fig — (TIFF) [file pbio.3003591.s011.tif]

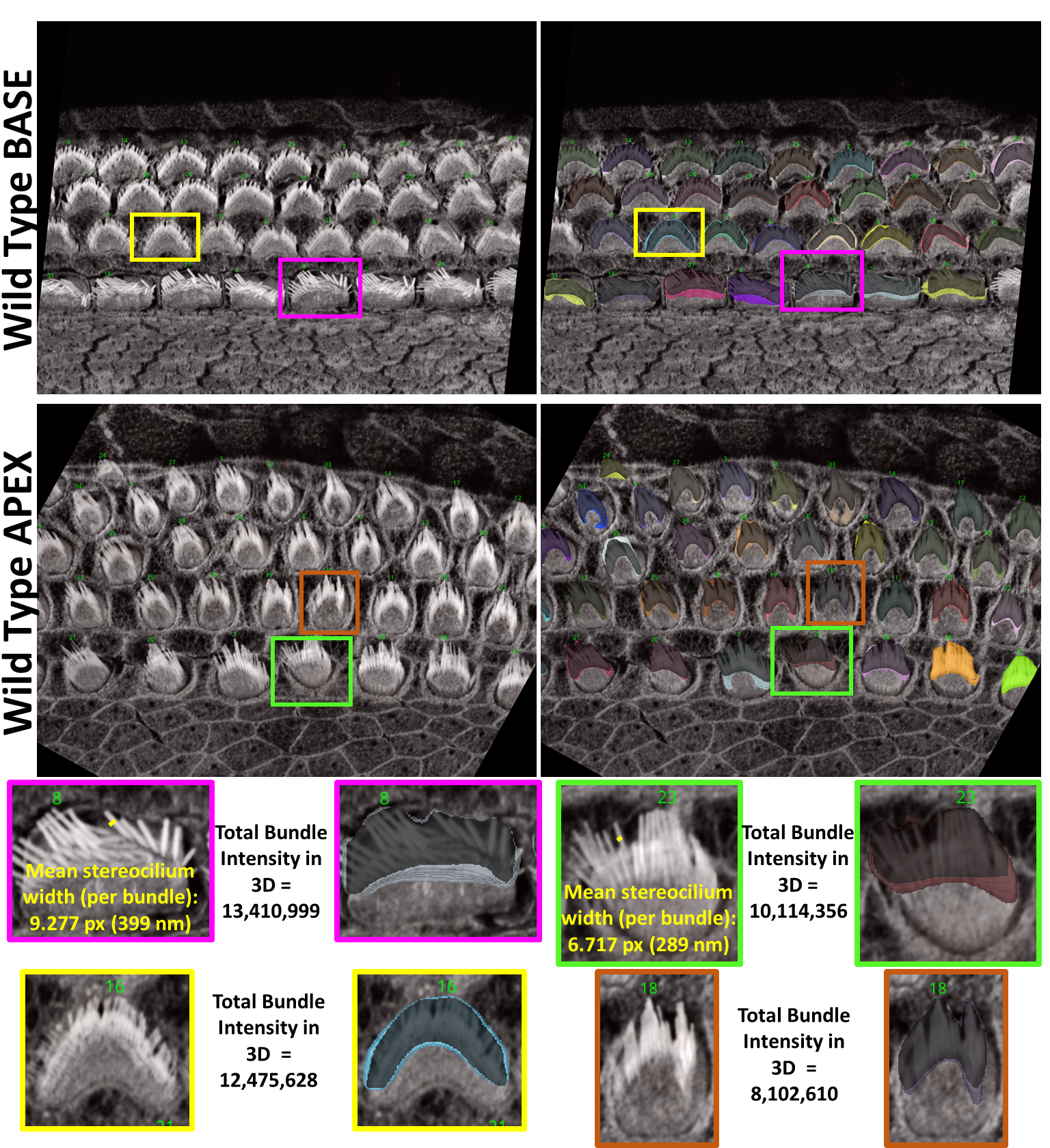

Supplement: S11 Fig — (TIFF) [file pbio.3003591.s012.tif]
