# Supplementary material for: VASCilia is an open-source, deep learning-based tool for 3D analysis of cochlear hair cell stereocilia bundles
Source: PLoS Biol. 2026 Jan 20;24(1):e3003591. doi: 10.1371/journal.pbio.3003591 (PMC12829968; doi:10.1371/journal.pbio.3003591)
Supplement: S1 Table — (PDF) [file pbio.3003591.s013.pdf]

Tonotopic_KO_WT_Class	mean_height	std_height	median_height	count
WT_Base_IHC	3.03	0.41	2.91	26
WT_Middle_IHC	3.27	0.46	3.24	28
WT_Apex_IHC	3.90	0.55	4.01	27
KO_Base_IHC	1.97	0.33	1.91	26
KO_Middle_IHC	2.07	0.24	2.05	26
KO_Apex_IHC	2.50	0.58	2.37	26
WT_Base_OHC	2.29	0.27	2.32	81
WT_Middle_OHC	2.52	0.40	2.47	85
WT_Apex_OHC	2.94	0.53	2.94	100
KO_Base_OHC	1.46	0.20	1.44	77
KO_Middle_OHC	1.54	0.23	1.51	88
KO_Apex_OHC	1.91	0.38	1.90	98

Table S1. Bundle height summary by genotype (WT/KO), tonotopic region (Base, Middle, Apex), and cell type (IHC/OHC), related to Fig 10A (top row). Values are in μm; each row reports mean, SD, median, and sample size (N). Row order matches the violin plot. Totals across all groups: IHC $N = 159$, OHC $N = 529$, overall $N = 688$.
