# Supplementary material for: VASCilia is an open-source, deep learning-based tool for 3D analysis of cochlear hair cell stereocilia bundles
Source: PLoS Biol. 2026 Jan 20;24(1):e3003591. doi: 10.1371/journal.pbio.3003591 (PMC12829968; doi:10.1371/journal.pbio.3003591)
Supplement: S2 Table — (PDF) [file pbio.3003591.s014.pdf]

KO_WT	Cell	Pair (level1–level2)	n1/n2	mean1	mean2	Δ (95% CI)	g	p_{adj} (Holm)	Status
WT	IHC	Base–Middle	26/28	3.03	3.27	−0.24 (−0.48, −0.01)	−0.56	0.0425	Significant
WT	IHC	Middle–Apex	28/27	3.27	3.90	−0.63 (−0.90, −0.36)	−1.23	5.35×10^{-5}	Significant
WT	IHC	Base–Apex	26/27	3.03	3.90	−0.87 (−1.14, −0.61)	−1.78	8.31×10^{-8}	Significant
WT	OHC	Base–Middle	81/85	2.29	2.52	−0.23 (−0.34, −0.13)	−0.68	1.86×10^{-5}	Significant
WT	OHC	Middle–Apex	85/100	2.52	2.94	−0.42 (−0.55, −0.28)	−0.87	1.42×10^{-8}	Significant
WT	OHC	Base–Apex	81/100	2.29	2.94	−0.65 (−0.77, −0.53)	−1.50	8.70×10^{-20}	Significant
KO	IHC	Base–Middle	26/26	1.97	2.07	−0.10 (−0.26, 0.06)	−0.35	0.209	Not significant
KO	IHC	Middle–Apex	26/26	2.07	2.50	−0.43 (−0.68, −0.18)	−0.95	0.00271	Significant
KO	IHC	Base–Apex	26/26	1.97	2.50	−0.54 (−0.80, −0.27)	−1.11	6.33×10^{-4}	Significant
KO	OHC	Base–Middle	77/88	1.46	1.54	−0.08 (−0.15, −0.01)	−0.37	0.0164	Significant
KO	OHC	Middle–Apex	88/98	1.54	1.91	−0.36 (−0.45, −0.28)	−1.15	3.69×10^{-13}	Significant
KO	OHC	Base–Apex	77/98	1.46	1.91	−0.45 (−0.53, −0.36)	−1.41	8.55×10^{-18}	Significant

Table S2. Pairwise tonotopic contrasts in bundle height by genotype (WT/KO) and cell type (IHC/OHC), related to Fig 10A (top row). Within each stratum we compare Base–Middle, Middle–Apex, and Base–Apex using Welch two-sided t-tests. Reported are sample sizes (n_1/n_2), group means (mean1/mean2, in μm), the difference $\Delta = \text{mean}_1 - \text{mean}_2$ with 95% CI, Hedges' g, and Holm-adjusted p-values. Negative Δ and g indicate level2 > level1. All contrasts are significant at $p_{adj} < 0.05$ except KO IHC Base–Middle.
