# Supplementary material for: VASCilia is an open-source, deep learning-based tool for 3D analysis of cochlear hair cell stereocilia bundles
Source: PLoS Biol. 2026 Jan 20;24(1):e3003591. doi: 10.1371/journal.pbio.3003591 (PMC12829968; doi:10.1371/journal.pbio.3003591)
Supplement: S3 Table — (PDF) [file pbio.3003591.s015.pdf]

Tonotopic_KO_WT_Class	mean_value	std_value	median_value	count
WT_Base_IHC	0.70	0.27	0.84	26
WT_Middle_IHC	0.24	0.13	0.18	28
WT_Apex_IHC	0.17	0.11	0.15	27
KO_Base_IHC	0.51	0.14	0.55	26
KO_Middle_IHC	0.33	0.09	0.32	26
KO_Apex_IHC	0.29	0.24	0.25	26
WT_Base_OHC	0.42	0.12	0.44	81
WT_Middle_OHC	0.16	0.09	0.13	85
WT_Apex_OHC	0.12	0.08	0.09	100
KO_Base_OHC	0.28	0.11	0.24	77
KO_Middle_OHC	0.26	0.09	0.27	88
KO_Apex_OHC	0.14	0.11	0.11	98

Table S3. Normalized fluorescence intensity (unitless) by genotype (WT/KO), tonotopic region (Base, Middle, Apex), and cell type (IHC/OHC), ordered to match the violin plot (related to Fig 10A (bottom row)). Each row reports the mean, SD, median, and sample size (N). Totals: IHC $N = 159$, OHC $N = 529$, overall $N = 688$.
