# Supplementary material for: VASCilia is an open-source, deep learning-based tool for 3D analysis of cochlear hair cell stereocilia bundles
Source: PLoS Biol. 2026 Jan 20;24(1):e3003591. doi: 10.1371/journal.pbio.3003591 (PMC12829968; doi:10.1371/journal.pbio.3003591)
Supplement: S4 Table — (PDF) [file pbio.3003591.s016.pdf]

KO_WT	Cell	Pair (level1–level2)	n1/n2	mean1	mean2	Δ (95% CI)	g	p_{adj} (Holm)	Status
WT	IHC	Base–Middle	26/28	0.702	0.241	$+0.461\,(0.342, 0.580)$	2.16	6.27×10^{-9}	Significant
WT	IHC	Middle–Apex	28/27	0.241	0.173	$+0.068\,(0.002, 0.133)$	0.55	0.0429	Significant
WT	IHC	Base–Apex	26/27	0.702	0.173	$+0.528\,(0.411, 0.646)$	2.51	3.98×10^{-10}	Significant
WT	OHC	Base–Middle	81/85	0.419	0.161	$+0.259\,(0.227, 0.290)$	2.53	2.57×10^{-34}	Significant
WT	OHC	Middle–Apex	85/100	0.161	0.123	$+0.038\,(0.013, 0.063)$	0.45	0.00258	Significant
WT	OHC	Base–Apex	81/100	0.419	0.123	$+0.297\,(0.266, 0.327)$	2.99	6.39×10^{-41}	Significant
KO	IHC	Base–Middle	26/26	0.512	0.329	$+0.183\,(0.116, 0.250)$	1.51	6.00×10^{-6}	Significant
KO	IHC	Middle–Apex	26/26	0.329	0.287	$+0.042\,(-0.062, 0.146)$	0.23	0.414	Not significant
KO	IHC	Base–Apex	26/26	0.512	0.287	$+0.225\,(0.113, 0.337)$	1.11	4.34×10^{-4}	Significant
KO	OHC	Base–Middle	77/88	0.277	0.258	$+0.0186\,(-0.0138, 0.0511)$	0.18	0.258	Not significant
KO	OHC	Middle–Apex	88/98	0.258	0.140	$+0.118\,(0.088, 0.148)$	1.12	1.32×10^{-12}	Significant
KO	OHC	Base–Apex	77/98	0.277	0.140	$+0.137\,(0.103, 0.171)$	1.20	1.07×10^{-12}	Significant

Table S4. Pairwise tonotopic contrasts for *normalized intensity* by genotype (WT/KO) and cell type (IHC/OHC), related to Fig 10A (bottom row). We report sample sizes (n_1/n_2), group means (mean1/mean2), difference $\Delta = \text{mean}_1 - \text{mean}_2$ with 95% CI, Hedges' g, and Holm-adjusted p-values. Positive Δ indicates level1 $>$ level2.
