# Supplementary material for: VASCilia is an open-source, deep learning-based tool for 3D analysis of cochlear hair cell stereocilia bundles
Source: PLoS Biol. 2026 Jan 20;24(1):e3003591. doi: 10.1371/journal.pbio.3003591 (PMC12829968; doi:10.1371/journal.pbio.3003591)
Supplement: S5 Table — (PDF) [file pbio.3003591.s017.pdf]

Block 1			Block 2			Block 3		
#	Manual	VASCilia	#	Manual	VASCilia	#	Manual	VASCilia
1	112.88°	111.10°	12	99.06°	95.34°	23	93.13°	91.16°
2	109.81°	110.56°	13	92.32°	95.23°	24	94.97°	94.14°
3	78.61°	76.35°	14	104.65°	102.91°	25	81.81°	81.49°
4	101.31°	95.34°	15	92.07°	92.69°	26	83.60°	87.44°
5	94.44°	97.28°	16	91.53°	87.61°	27	91.46°	90.36°
6	93.99°	93.89°	17	99.88°	99.33°	28	91.01°	90.81°
7	98.62°	94.76°	18	77.42°	75.48°	29	89.46°	89.60°
8	87.31°	88.01°	19	100.13°	97.38°	30	92.29°	92.47°
9	88.23°	90.00°	20	91.15°	89.55°	31	104.42°	102.40°
10	99.05°	98.39°	21	92.70°	91.60°	32	90.53°	88.45°
11	113.96°	113.20°	22	83.06°	86.16°	33	88.57°	90.35°
**Summary —** Mean (Manual/VASCilia): 94.04 / 93.35; Median: 92.31 / 92.47; SD: 8.83 / 8.44								

Table S5. Per-cell orientation angles measured manually in Fiji and compared with VASCilia (related to Fig 14)
