# Supplementary material for: VASCilia is an open-source, deep learning-based tool for 3D analysis of cochlear hair cell stereocilia bundles
Source: PLoS Biol. 2026 Jan 20;24(1):e3003591. doi: 10.1371/journal.pbio.3003591 (PMC12829968; doi:10.1371/journal.pbio.3003591)
Supplement: S6 Table — (PDF) [file pbio.3003591.s018.pdf]

Block1			Block2		
#	Manual	VASCilia	#	Manual	VASCilia
1	103.75°	99.68°	10	91.18°	90.53°
2	87.73°	89.43°	11	85.14°	84.44°
3	88.17°	87.95°	12	89.42°	86.52°
4	72.48°	80.68°	13	80.01°	85.75°
5	103.04°	98.58°	14	94.86°	89.51°
6	79.96°	76.09°	15	89.39°	88.89°
7	84.80°	85.24°	16	82.46°	84.13°
8	85.56°	80.89°	17	91.14°	85.75°
9	85.87°	82.95°			
**Summary —** Mean (Manual/VASCilia): 87.94 / 86.88; Median: 87.73 / 85.75; SD: 7.80 / 5.90					

Table S6. Comparison of orientation angles measured manually in Fiji and by VASCilia, displayed in two horizontal panels (related to Fig 15).
