# Supplementary material for: VASCilia is an open-source, deep learning-based tool for 3D analysis of cochlear hair cell stereocilia bundles
Source: PLoS Biol. 2026 Jan 20;24(1):e3003591. doi: 10.1371/journal.pbio.3003591 (PMC12829968; doi:10.1371/journal.pbio.3003591)
Supplement: S7 Table — (PDF) [file pbio.3003591.s019.pdf]

Block A			Block B			Block C		
#	Manual	VASCilia	#	Manual	VASCilia	#	Manual	VASCilia
1	1.603	1.696	6	2.252	2.179	11	1.989	1.986
2	1.629	1.715	7	2.086	2.009	12	1.703	1.741
3	1.855	1.858	8	2.832	2.816	13	1.313	1.277
4	1.711	1.634	9	2.035	1.965	14	1.635	1.787
5	1.193	1.218	10	2.216	2.249	15	1.333	1.400
					**Mean / SD**	**Mean**	**1.826**	**1.835**
						**Std Dev**	**0.426**	**0.405**
					**Paired t-test p-value**	**0.609**		
					**Wilcoxon signed-rank p-value**	**0.720**		

Table S7. Per-cell length values from human-annotated ground truth for *Eps8* KO mice using Fiji and VASCilia for 15 cells. The comparison showed no statistically significant difference between the two methods. Specifically, the mean stereocilia length was 1.826 µm for Fiji and 1.835 µm for VASCilia, with standard deviations of 0.426 µm and 0.405 µm, respectively. A Wilcoxon signed-rank test and a paired t-test both confirmed the absence of significant differences.
