# Supplementary material for: VASCilia is an open-source, deep learning-based tool for 3D analysis of cochlear hair cell stereocilia bundles
Source: PLoS Biol. 2026 Jan 20;24(1):e3003591. doi: 10.1371/journal.pbio.3003591 (PMC12829968; doi:10.1371/journal.pbio.3003591)
Supplement: S8 Table — (PDF) [file pbio.3003591.s020.pdf]

Block A			Block B			Block C		
#	Manual	VASCilia	#	Manual	VASCilia	#	Manual	VASCilia
1	1.964	1.948	6	1.777	1.803	11	1.658	1.684
2	1.873	1.879	7	1.756	1.790	12	1.430	1.248
3	2.139	2.105	8	2.754	2.739	13	1.186	1.082
4	2.260	2.356	9	1.475	1.592	14	0.866	0.886
5	1.735	1.955	10	2.117	1.948	15	1.697	1.751
					**Mean / SD**	**Mean**	**1.779**	**1.784**
						**Std Dev**	**0.455**	**0.468**
					**Paired *t*-test *p*-value**	**0.851**		
					**Wilcoxon signed-rank *p*-value**	**0.639**		

Table S8. Per-cell length values from human-annotated ground truth for *Cdh23*$^{-/-}$ mice using Fiji and VASCilia for 15 cells. The comparison showed no statistically significant difference between the two methods. Specifically, the mean stereocilia length was 1.779 µm for Fiji and 1.784 µm for VASCilia, with standard deviations of 0.455 µm and 0.468 µm, respectively. A Wilcoxon signed-rank test and a paired t-test both confirmed the absence of significant differences.
