# Supplementary material for: VASCilia is an open-source, deep learning-based tool for 3D analysis of cochlear hair cell stereocilia bundles
Source: PLoS Biol. 2026 Jan 20;24(1):e3003591. doi: 10.1371/journal.pbio.3003591 (PMC12829968; doi:10.1371/journal.pbio.3003591)
Supplement: S9 Table — (PDF) [file pbio.3003591.s021.pdf]

Block A			Block B			Block C		
**ID**	**Manual**	**VASCilia**	**ID**	**Manual**	**VASCilia**	**ID**	**Manual**	**VASCilia**
cell8	86.32°	88.17°	cell13	88.32°	90.00°	cell18	101.30°	101.31°
cell7	83.26°	86.68°	cell14	102.46°	96.71°	cell20	104.85°	109.03°
cell24	95.98°	92.49°	cell15	60.09°	68.20°	cell48	140.98°	139.09°
cell6	90.68°	90.00°	cell9	109.07°	103.39°	cell33	140.20°	138.99°
cell5	91.56°	91.61°	cell11	119.81°	117.98°	cell26	107.27°	104.04°
cell4	87.22°	89.26°	cell44	113.11°	112.99°	cell29	122.89°	123.69°
cell1	71.19°	75.23°	cell45	135.39°	131.28°	cell32	77.60°	77.91°
cell3	85.28°	84.35°	cell42	92.95°	92.39°	cell38	29.47°	31.18°
cell2	74.90°	79.00°	cell34	98.97°	96.77°	cell36	80.41°	82.03°
cell39	129.93°	125.54°	cell35	107.80°	103.24°	cell40	126.73°	128.88°
cell27	146.07°	151.19°	cell30	121.94°	121.61°	cell37	117.13°	115.46°
cell31	123.36°	128.99°	cell19	119.58°	124.22°	cell47	64.80°	66.80°
cell28	90.53°	90.00°	cell16	90.43°	88.98°	cell41	116.55°	114.90°
cell25	110.79°	109.44°	cell21	92.18°	92.39°	cell43	122.59°	122.35°
cell22	151.15°	149.66°	cell12	75.11°	78.69°	cell46	107.57°	106.86°
cell17	136.45°	135.00°						
						**Mean**	**103.090°**	**103.222°**
						**Std Dev**	**24.940**	**24.061**
						**Paired t-test p-value**	**0.783**	
						**Wilcoxon signed-rank p-value**	**0.965**	

Table S9. Per-bundle orientation values (degrees) for a PCP-deficit mouse cochlear dataset[41], see Fig S4, measured in Fiji (manually) and with VASCilia (automated). A paired t-test and a Wilcoxon signed-rank test indicate no significant difference.
