# Supplementary material for: VASCilia is an open-source, deep learning-based tool for 3D analysis of cochlear hair cell stereocilia bundles
Source: PLoS Biol. 2026 Jan 20;24(1):e3003591. doi: 10.1371/journal.pbio.3003591 (PMC12829968; doi:10.1371/journal.pbio.3003591)
Supplement: Attachment — Submitted filename: Response to Reviewers.docx [file pbio.3003591.s023.docx]

NOTE: Due to the length of some of our responses, we have generated “tl;dr” (“too long, didn’t read”) summaries of our response above each longer response, highlighted in yellow. Full responses are in **blue.**

**Reviewer #1:** **This would certainly be a useful tool for hair cell biologists and the paper gives a thorough account of its development and features. However, the paper is long and very dense and I think a lot of the text could be moved onto their website as files to read when installing or using the program. I think the paper should serve more as a proof of principle for the different features, however some of that kind of data is lacking, especially for the measurement features (Figs 8-13).**

**tl;dr:** We agree and have streamlined the manuscript by moving detailed user-guide material to our online documentation, focusing the main text on scientific proof-of-principle and key findings.

**Location of Changes:** The "how-to" descriptions of the VASCilia workflow and UI have been removed from the main text. The revised Discussion (Section 2) now synthesizes scientific findings rather than user instructions. All UI screenshots have been moved to the online documentation.

**We thank the reviewer for the thoughtful assessment. We agree that user-guide/utility material is better suited to online documentation and that the main text should emphasize proof-of-principle. Accordingly, we moved step-by-step workflow descriptions, button/layer details, file names, and other usage instructions out of the main text and into the Supplementary Materials and the VASCilia documentation (link provided in the manuscript). We also refocused the Discussion away from “how to use” material so that it now synthesizes the scientific and quantitative findings.**

**We also have removed all screenshots (in the supplementary materials) that describe how to use the software and relocated them to our online documentation. All workflow steps and interface buttons are now documented with step-by-step screenshots at {https://ucsdmanorlab.github.io/Napari-VASCilia/}.**

**Although we removed utility prose, we incorporated new analyses and figures to address the reviewer’s requests. The scope of this work, including five machine-learning modules, multiple measurement components, and two biological case studies, requires presentation of essential results, methods, and controls to ensure reproducibility and to establish proof-of-principle. We therefore do not believe that further substantive condensation is feasible without compromising clarity or rigor.**

**We now explicitly highlight that, once 3D instance segmentation is obtained, VASCilia automatically extracts height, depth-matched phalloidin intensity, orientation, tonotopic position, row identity, 2D and 3D measurements, and texture features across large cohorts. This compresses analyses that typically require months of manual effort into hours. We also added a dedicated Limitations and Future Work paragraph in the Discussion section.**

**What I think could be improved is as follows:**

**1) I would have loved to test out this plugin but was not able to get it installed following instructions in the ReadMe document that they direct you to in the paper. I also could not access the other documents in the github site. In order for this to be more user-friendly there has to be better installation instructions provided in the paper including the system requirements needed on the computer. I know of one other person who eventually got it installed on their PC but was unable to run the example files they provide and the software was unable to open their confocal images without crashing. I think without clear user-friendly instructions for the installation this will not be very usable for most labs. Perhaps a detailed tutorial written for one of the demo files would be useful for guiding the use of the plug-in.**

**tl;dr:** We apologize for the issues and have released a new version (VASCilia 1.4.0) with a simplified two-command installation process and a comprehensive, step-by-step online guide.

**Location of Changes:** The new, simplified installation instructions and system requirements are now available in the dedicated online guide, which is linked in your response.

Thank you so much for taking the time to try VASCilia and for the detailed, constructive feedback. We are sorry you ran into installation and documentation issues. We have worked to improve every aspect of VASCilia:

We’ve just released **VASCilia 1.4.0**, which directly addresses the points you and other reviewers raised and also incorporates requests from other labs; you will find the new update and features in the release tag.

Most importantly, we include, as you suggested a **Clear, step-by-step installation guide + system requirements**: [https://ucsdmanorlab.github.io/Napari-VASCilia/installation.html](https://ucsdmanorlab.github.io/Napari-VASCilia/installation.html?utm_source=chatgpt.com)
 We verified these steps on three different workstations.

Now, VASCilia installation needs just two commands after you create the Conda environment.

conda create -y -n napari-VASCilia -c conda-forge python=3.10

conda activate napari-VASCilia

1. pip install --extra-index-url https://download.pytorch.org/whl/cu113 torch==1.12.1+cu113 torchvision==0.13.1+cu113 torchaudio==0.12.1+cu113
2. pip install napari-vascilia

napari

The first command installs the GPU dependencies; the second installs VASCilia. Please verify that your system meets the prerequisites listed in the Specifications section to avoid installation issues. Since VASCilia uses 5 deep learning models, we are unable to publish model weights with the package; this requires the user to download the model weights from the specified link and adjust the paths in the config file, which will be automatically created after the launch of the plugin in napari. The user also needs to download our samples to test the software, and specify the path in the config file to store the intermediate results. We tried to make the installation as simple as possible, but we cannot eliminate the first-time effort for specifying the paths in the config file. All these step-by-step details are now provided on the installation page.

**2) In Figure 4 (and the other figures), it is unclear to me how the bundle segmentations appear in 3D or how far they extend into the z plane. It seems that most of the images are fairly shallow stacks of flattened hair bundles and judging from Figure 4 (and from figure 28) the bundle segmentation is fairly shallow and uniform within the z-axis. What happens with images where the bundle is not flattened and varies in shape more throughout the z-axis? Also, does the segmentation extend into the cuticular plate at all or is there cell-to-cell variability in how much cuticular plate is included? The phalloidin intensities in the cuticular plate and the stereocilia can be quite similar at certain timepoints which may make segmentation of only the bundle more difficult. The segmentations in Figure 6 appear to be for more upright bundles. It would be good to show one of these close up at different rotations to see the segmentation at xz and yz planes and to keep the raw actin signal separate to see how the segmentation aligns with the bundle in all planes.**

**tl;dr:** We have added a new supplementary figure showing detailed 3D segmentation, including per-slice overlays and orthogonal views, which confirms that our method accurately follows the stereocilia signal and excludes the cuticular plate.

**Location of Changes:** A new Supplementary Figure S1 has been added. The Discussion (Section 2, page 17) has been updated to reference this new figure and explicitly state its findings.

Thank you for your thoughtful comment. We apologize for the confusion and acknowledge that the original manuscript did not include examples showing the 2D segmentation across all Z-layers. To clarify, the apparent shallowness of the segmentation in the Z-axis is not uniform across all cells. It depends significantly not just on the orientation of the bundle relative to the imaging plane, but also on the visibility and continuity of the phalloidin signal across Z-planes, which can vary between cells due to differences in imaging conditions (especially, the Z-step) and stereocilia morphology.

Importantly, our annotation protocol captures only the stereocilia bundle. Segmentation does not extend into the cuticular plate and begins only where a clear stereocilia signal is present. This avoids ambiguity when phalloidin intensity in the cuticular plate is strong.

To address the raised concern, we have now included Figure S1, which provides two representative examples from the P21 apical region (APEX region). For each example, we show:

·	All 2D layers of the raw image and their corresponding segmentation overlays.

·	A 3D view of the segmentation overlaid on the raw signal.

·	More views in different rotations and side projections.

This additional figure clarifies the 3D bundle architecture and the segmentation’s extent along the z-axis, illustrating how mask depth follows bundle morphology (upright vs. flat).

We selected this example first because the bundle is fully rotated into the Z-plane (upright), and second because it exhibits a strong phalloidin signal in the cuticular plate. Despite this intense background signal, the segmentation algorithm successfully detected only the stereocilia bundles. VASCilia was able to segment all layers in Z where the bundle was present, stopping precisely where the stereocilia signal entered the cuticular plate (layers 1-5).

**Another example from the same apical region of a P21 cochlea, highlighting a challenging flattened stereocilia bundle.**

This bundle is noticeably flat, **heavily overlaps** with neighboring bundles, and is surrounded by **intense background phalloidin signals**. Despite these challenges, the segmentation algorithm precisely detects only the stereocilia bundles. Notably, the mask appears in layer 6, where the stereocilia become clearly visible, but not in layer 4 and 5, where the stereocilia signal remains unclear although with a strong background phalloidin signal. This selective detection illustrates the algorithm’s precision and robustness in identifying features only when in focus.

**We’ve added this to the Discussion section in the paper “To highlight variability in segmentation depth as a function of bundle orientation (upright vs. flat) and phalloidin-signal continuity, and to show that the segmentation excludes the cuticular plate, Supplementary Fig. S1 (a,b) presents two bundles from a representative apical P21 cochlear stack, including all z-planes with and without overlays; orthogonal slice views (XY, XZ, YZ); and 3D volume renderings”**

**3) For the height measurements in Figure 8, how are the two observers performing the measurements? I'm assuming it's by marking the top and base of the tallest stereocilia within the z-stack? This approach to height measurement, especially given the varying orientation of these bundles, will likely add quite a lot of variability. Can you compare these measurements to another approach?**

**tl;dr:** We have clarified the manual 3D measurement method (marking base and tip in Fiji) and explain that VASCilia's automated analysis serves as the direct, high-throughput comparison, which shows strong statistical agreement.

**Location of Changes:** The manual measurement protocol is now explicitly detailed in Methods (Section 3.7) under the new sub-heading "Manual observer method".

Thank you for raising this important point regarding height measurement variability. To clarify, the two observers performed the height measurements using *Fiji*. For each bundle, they identified a point at the base of the tallest stereocilium and then scrolled through the Z-stack to locate the tip of the same stereocilium. The 3D Euclidean distance between these two points was then calculated based on the (x, y, z) coordinates and the physical resolution of the image.

To minimize bias, we split the work across two independent teams. Team 1 generated ground-truth heights by marking base and tip in Fiji/ImageJ and computing 3D Euclidean distances. Team 2, which never saw those measurements, created stereocilia masks and trained the segmentation model; no length labels were used for training. Our pipeline then derives height from the 3D geometry of the predicted masks. Thus, the close agreement with the independent Fiji measurements is exactly what the method is designed to achieve. This convergence shows that the segmentations capture bundle extent with sufficient fidelity to recover length, providing an unbiased and scalable alternative to manual measurement.

Importantly, we recognize that this approach of manual length measurements using Fiji is time-consuming and becomes impractical at scale. One key challenge is that while scrolling through the stack, it can be difficult to consistently track and remember the exact stereocilium being measured. As a result, the observers often have to double-check their selections and verify the measurement multiple times before finalizing it, which further adds to the manual burden.

VASCilia was created precisely to address this issue. We developed our own 3D segmentation-based measurement tool that simulates the annotators’ workflow for identifying the top and bottom of the longest stereocilium for each bundle in an automated and consistent manner. This approach enables us to scale the analysis to a much larger number of cells while maintaining precision. To our knowledge, there is currently no existing tool that allows for accurate and efficient height measurement of stereocilia bundles from 3D image stacks in this way. Thus, our tool fills a gap in the field by providing a reproducible, geometry-aware solution tailored to this biological structure.

**We’ve added the following paragraph to Section 3.7 to clarify how manual bundle height is calculated:**

**The height of stereocilia was manually measured in Fiji (ImageJ). For each bundle, an observer (i) identified the tallest stereocilium, (ii) placed a marker point at its base (x,y,z), (iii) scrolled through the z-stack to the tip of the same stereocilium to place a second marker (x,y,z), and (iv) calculated the 3D Euclidean distance between the base and tip using the known voxel dimensions (the same formula as above). Each bundle was independently measured by two observers.**

**4) What are the four datasets in figure 8? Are they all from the same age and tonotopic region? The heights are averaged in Table 3 so I am assuming they are but the values for each dataset are quite different. If they are from the same area, then it appears the orientation of the stereocilia has a huge effect on the height measurement. This would make sense if the amount of stereocilia length in the z-axis rather than xy varies from cell to cell. The approach seems most viable only for the more flattened bundles.**

**tl;dr:** We clarified that the variability is biological, as the data includes WT, Eps8 KO with and without AAV-mediated gene therapy treatment, IHC, and OHC samples. Our 3D measurement is robust to orientation, and we've added a new figure to show the source images.

**Location of Changes:** A new Supplementary Figure S2 shows the source z-stacks. The caption for Table 4 now refers to this figure. Additionally, a new Supplementary Figure S3 and corresponding text in the Discussion (Section 2, page 17) demonstrate the rotation-invariance of the 3D length measurement.

We apologize for not providing more detail on the datasets used for this figure, which is meant to illustrate the variability between VASCilia and human experts, and not to distract from that key focus with the biology, which is outside the scope of this manuscript. However, we also aimed to ensure our analyses were not overfit to specific conditions, and thus chose to include more than one experimental/biological condition.

The four datasets shown in Figure 8 are from Z-stacks selected for manual stereocilia height measurements. All stacks originate from the apical region of the cochlea and represent two wild-type P5 mice (Datasets 1+2) and two full-length Eps8 KO (Datasets 3+4) P5 mice that had undergone ANC80L65 AAV-GFP-EPS8 injection at P0 (as described in Jeng et al., 2022). Within each dataset, cells for measurement were randomly chosen to include both IHCs and OHCs. The datasets also offer both, transduced and untransduced hair cells, since AAV transduction was not 100% efficient. In short, the differences observed are due to the variability in biological conditions (i.e. IHC vs OHC, WT vs. KO, KO with vs without AAV), and not due to imaging/computational technicalities. To provide clarity and transparency, we have now included visualizations of all four full stacks (datasets) in Supplementary Figure S2, with the specific measurement crops outlined in red. These are the same regions shown in Table4.

We agree that both the orientation and the extent of bundle elongation along the z-axis can influence height measurements, and this variability affects both manual and automated methods. However, performing such measurements manually across hundreds or thousands of cells is nearly impossible due to the time and effort required. This is precisely where automated measurements offer a major advantage—by enabling large-scale, consistent quantification that helps to average out minor variations, revealing robust biological patterns.

**We’ve updated the caption of Table 4 to ‘Data set crops that are associated with the computations used in Table 3, See supplementary figure S2, where all four complete datasets are visualized for reference.’**

To address the comment stating: **‘The approach seems most viable only for the more flattened bundles.’** We have also included an additional example demonstrating that the plugin accurately measures bundle length regardless of whether the bundle is **flattened or not**. This example has been included in the supplementary materials.

**As a result, we added the following to the Discussion section:
‘Beyond agreement with experts, a key methodological advantage is that our length metric is rotation-invariant because it traces bundle extent through the z-stack rather than relying on projected height. As shown in Supplementary Figure S3, rotated OHC1 bundles traverse more layers (depth =10) than the straighter OHC2/3 bundles (depth =5), yet the computed lengths are comparable. For example, OHC1 cell bundles with IDs 17 and 18 measure 1.65--1.69 µm and that of cell bundle 16 is 1.75 µm, comparable to OHC2 cell bundle 38 at 1.71 µm. The apparent visual foreshortening effect of rotated bundles reflects viewing angle, not true length; the increased z-plane depth afforded by the 3D imaging data accounts for these angle differences.’**

**5) The comments in the text about the time needed for height measurements in Fiji versus in the program does not seem accurate. It is likely that the user will have to go through and refine the base and top points that the program provides or at least would have to double check that they were accurate. This would significantly minimize the time difference.**

**tl;dr:** Our time estimate is accurate and conservative; most bundles do not require refinement, and when they do, the process is very fast in VASCilia's 3D viewer compared to the laborious manual process in Fiji.

**Location of Changes:** The revised text in Results (Section 1.4.1, page 6) clarifies the time estimate, and your response document provides extensive details on the fully manual Fiji workflow that supports the claim.

We would like to clarify that the manual height measurements performed in Fiji were completely independent from the automated program. VASCilia was not installed or used by any of the observers at the time of measurement. In fact, users carried out the measurements in parallel, each working independently and without access to one another’s results, to avoid bias.

The time we reported reflects the actual manual annotation effort under these conditions.

We confirmed that the reported time ***in Fiji*** includes selecting the base and tip of the stereocilium using the multipoint tool functionality, and verifying the selection—often twice—due to the challenge of tracking the correct stereocilium while scrolling through the Z-stack. The measurements were then saved to a CSV file, opened separately to calculate the 3D Euclidean distance using the distance formula, and used to manually record the results in a shared Google document. The time and effort for this workflow and experience was identical between independent observers in the Fiji environment. This entire process is extremely slow and laborious.

In contrast, our ***VASCilia*** automated pipeline handles all preprocessing, measurement saving, and distance calculation seamlessly, eliminating this manual burden. We did mention in the manuscript that it does sometimes benefit from some minimal refinement for several cells and double-checking, which we included in our total time estimate, which is at most 5 minutes to refine occasional errors and double-check the visualization, however it is usually much less than that.

We routinely use VASCilia in our ongoing inner ear research projects to generate statistical data. This has enabled us to obtain height measurements from thousands of cells, an analysis that would be virtually impossible to conduct manually due to the time and effort required.

**6) While the measurements between the program and observers are not different (Table 3), they are all highly variable judging by the standard deviations in the table. This result likely derives from the variability in the bundle positions in the images and the way in which their calculation method intersects with this variability, which would apply to both program and observer calculations. The SD is about 25% of the mean length making finding significant lengths changes between conditions quite challenging. For the data in Figure 9a, one can see that the variability is quite large across all conditions and I'm not sure what level of significance could be measured. I would like to see a table of these values that include mean and standard deviation as well as any statistical tests.**

**tl;dr:** We agree the variability reflects biological heterogeneity. We have now added supplementary tables with full descriptive statistics and pairwise statistical tests, which confirm significant tonotopic gradients for both height and intensity (this also addresses comment (7) below regarding intensity variability)

**Location of Changes:** Supplementary Tables S1, S2, S3, and S4 have been added with full statistical details. The new Results Section 1.4.3 ("Case Study 1") now refers to these tables. Figure 9b (intensity) was also re-analyzed using a depth-controlled method for improved clarity.

We agree that differences in variability in the bundle positions, bundle orientation, and layer depth may result in SD differences- this is in part due to the inherent differences in xy vs. z resolution in 3D confocal imaging. However, our segmentations are 3-dimensional, and as shown in our examples above, our tool is able to segment the top and bottom of the bundles even when they are oriented orthogonal to the imaging plane. Thus, while the accuracy may vary with bundle orientation, the overall trend will be robust, which is illustrated by the statistically significant differences in bundle heights between regions and genotypes.

Furthermore, the biological variability will also be significant; for these measurements, we combined all OHCs together, when in fact it is known that each row of OHCs have slightly different properties. Also, our “apex” vs “middle” vs “base” categories still leave lots of room for biological variability depending on precise location of where each image was taken within the cochlea.

Finally, even with IHC bundles within the **same image (e.g. the exact same tissue region, exact same animals)**, we may observe large variability due to the occasional occurrence of a single abnormally long stereocilium. See for example Cell #46 in Figure 8 (copied below too). In this example, both VASCilia and the two human experts rated this bundle **as ~1 micrometer longer** than the two IHCs immediately adjacent. This is in spite of the fact that these bundles at low magnification appear to be virtually identical.

Importantly, the differences in these measurements were highly consistent between VASCilia and the two human experts. This is in fact our criteria for success of our (Manor Lab) deep learning-based algorithms: if the automated process performs within the range of inter-human expert measurements, then we believe the workflow is suitable for deployment, given the enormous cost-savings provided by these automations.

In response to the request, we provide (i) a descriptive table with mean, SD, median, and N for each group and (ii) a table of pairwise tonotopic contrasts. Within each genotype × cell-type stratum (WT IHC, WT OHC, KO IHC, KO OHC). We compared Base vs Middle, Middle vs Apex, and Base vs Apex using Welch’s unequal-variance two-sided t-tests to test neighboring steps along the cochlea. For each comparison we report Δ (mean₁ − mean₂), 95% CIs, Hedges’ g (small ≈ 0.2, medium ≈ 0.5, large ≥ 0.8), and Holm-adjusted p-values (p_adj < 0.05 = significant). Note: the sign of g follows Δ; negative values indicate level2 > level1.

**Results summary.**

WT (IHC and OHC): clear tonotopic increase (Base < Middle < Apex); all adjacent steps are significant with medium–very large effects (largest for Base–Apex).

KO OHC: also monotonic (Base < Middle < Apex); all adjacent steps are significant, with a smaller Base–Middle step.

KO IHC: partial gradient—Base ≈ Middle (not significant), but Middle → Apex and Base → Apex are significant with large effects.

*Note on “Middle”*: It’s common to see weaker or inconsistent significance for comparisons involving Middle, because those samples can fall near the Base/Middle or Middle/Apex boundary; the biological gradient is continuous, so “Middle” often blends toward one neighboring region.

**We’ve added this text to section 1.4.3 and the two tables in the supplementary materials**

**Supplementary tables S1 and S2 summarize bundle-height distributions (mean, SD, median, N) and the pairwise tonotopic contrasts. They show a robust Base→Middle→Apex increase in WT (IHC/OHC) and KO OHC, with the only non-significant step being Base vs. Middle in KO IHC (Holm-adjusted tests).**

**We also uploaded all the csv files related to this study**

**7) The total intensity measurements in figures 9 and 10 are also incredibly variable. For Figure 9, I would also like to see a table of these values that include mean and standard deviation as well as any statistical tests. I don't think the authors can make any claims about the correlation between height and actin intensity without showing the statistics.**

**Short Answer: Thanks, we agree that phalloidin intensity is variable, reflecting biological heterogeneity. We added Supplementary Table S3 (mean, SD, N by group) and re-ran the analysis with a fixed bundle depth to reduce sampling-depth effects (we explain what “fixed bundle depth” means in our response to the next comment). With this, the Base→Apex decrease is more clear. In terms of significance (Table 10), all pairwise contrasts are significant except two, KO IHC Middle–Apex and KO OHC Base–Middle, both involve the Middle region, which often includes bundles leaning toward either neighbor, making the contrast less pronounced. Furthermore, as highlighted throughout the manuscript, KO cells are overall much shorter, and therefore have smaller absolute differences between regions.**

**Long Answer:**
 We thank the reviewer for this important observation. We agree that total phalloidin‐labeled actin intensity can be variable, reflecting biological heterogeneity in hair-bundle morphology and differences in sample preparation, staining and imaging conditions. To improve transparency, we have added a summary table (Table 9) reporting the mean, standard deviation, and sample size for each group shown in Figure 9. In addition, to reduce technical variation related to sampling depth, we re-analyzed the data using a constant bundle depth (a new option in our code in which **the sum restricted to the top (n) layers, where (n) is the largest depth shared by all bundles being compared within each 3D dataset**). This analysis yields a clearer Base→Apex tonotopic gradient than our previous analyses in which bundle depth varies.

**New Figure using Mode2:**

Following exactly the same steps in the previous comment, we get this table:

1. WT / IHC: Base > Middle > Apex. All adjacent steps are significant.
2. WT / OHC: Base > Middle > Apex. All adjacent steps are significant
3. KO / IHC: Base > Middle, but Middle ≈ Apex (not significant). Base > Apex is significant.
4. KO / OHC: Base ≈ Middle (not significant), and both > Apex (significant).

Overall: WT shows a clear decrease in normalized intensity from Base→Apex; KO shows a weaker gradient (one adjacent step is not significant in each KO cell type).

Note on the “Middle” region: it’s very typical to see weaker or inconsistent significance for comparisons involving Middle because those samples can lie near the Base/Middle or Middle/Apex boundary. Since the biological gradient is continuous, “Middle” often includes bundles whose characteristics are closer to Base or to Apex, which blunts the contrast.

**We’ve added this text to section 1.4.3 and the two tables in the supplementary materials**

**See Supplementary Tables S3 and S4, which report descriptive statistics (mean, SD, median, N) and pairwise contrasts (Delta, 95% CI, Hedges’ *g*, Holm-adjusted p-values); significant comparisons are indicated.**

**Raw values for each of the 12 groups shown here are saved as separate CSV files and uploaded with the other materials. The two tables here added to the supplementary material section**

**The code and data for generating this case study is available in the new** released version **VASCilia 1.4.0**

[**Napari-VASCilia/downstream_analysis/WTvsKO_casestudy.py at main · ucsdmanorlab/Napari-VASCilia**](https://github.com/ucsdmanorlab/Napari-VASCilia/blob/main/downstream_analysis/WTvsKO_casestudy.py)

**8) In figure 10, one can see some cell-to-cell variations in bundle segmentation that could contribute to intensity variation in the plot. Could you show the segmentation from another angle? It seems likely that there could be variation in the amount of cuticular plate actin that would be included in each segmentation which would affect these intensity measurements. How do these values compare to another approach for actin intensity measurement?**

**tl;dr:** We've revised Figure 10 to show that intensity variation is primarily driven by biological factors and bundle depth, not segmentation errors. We now offer three depth-controlled analysis modes and provide per-slice examples to demonstrate that the cuticular plate is correctly excluded.

**Location of Changes:** Figure 10 has been completely revised to include three analysis modes and per-layer examples. A clarifying paragraph has been added to the Discussion (Section 2, page 18).

**Long Answer:** We appreciate the reviewer’s thoughtful observation about cell-to-cell segmentation differences and their impact on the intensity plots in Figure 10. Because we realized that depth is a major source of variability, we now provide three analysis modes (shown in Figure 10): (1) total raw intensity over all layers; (2) total raw intensity over a fixed common depth (top n layers shared by all bundles); and (3) a depth-padded total, in which shallower bundles are padded to n layers by repeating their bundle’s weakest-intensity slice layer.

**We also revised normalization to max-scaling (value ÷ per-plot maximum) instead of min–max, which avoids forcing any bundle to zero (e.g., bundle 38) while preserving relative differences. Values are reported as raw (pre–background-subtraction) phalloidin intensity rather than with background background-subtraction in the submitted version.**

To make inclusion/exclusion explicit, we extended Figure 10 with four illustrative cells (two IHC, two OHC), each shown as six consecutive z-slices plus the final 3D rendering. These examples are matched in depth, so differences reflect segmentation boundaries and the underlying actin signal rather than sampling depth. They also show that total intensity varies with bundle size, texture, height, and filamentous-actin density; depth differences further increase variance.

Rather than adding another 3D viewing angle, which can still be ambiguous, we show the full slice context. This makes clear how much of the apical region (bundle vs. cuticular plate) is included. Our model was trained to exclude cuticular plate signal unless stereocilia extend into that region, and this is evident in the 2D views.

For context, the most common approach in the literature is 2D maximum-intensity projection (MIP) with manual masks, which collapses depth and often mixes cuticular-plate signal into the measurement. By contrast, our 3D volume integration quantifies stereocilia voxels only, yielding more accurate and less variable estimates.

Finally, there is no standardized 3D ground-truth dataset for phalloidin intensity in stereocilia bundles. We view this as an important area for future work—e.g., with STED or expansion microscopy—and hope this manuscript encourages data sharing to enable such benchmarking. Practically, the same 3D pipeline is already being used in our lab for other markers (e.g., EPS8, MyoXVa); the measurement is channel-agnostic and configurable via a single parameter.

**We’ve added this paragraph to the paper to the Discussion.**

“**One potential limitation when measuring total intensity between bundles is the variability in segmentation depth across cells, which can arise from differences in fluorescence signal strength (phalloidin here), imaging depth, or biological heterogeneity. This variability may cause some bundles to appear shallower, potentially affecting their total intensity measurements. Although such differences tend to average out when large numbers of cells are analyzed, minimizing their impact on overall trends and group comparisons, we also provide a dedicated option for researchers who wish to control for this factor. Specifically, VASCilia outputs a CSV file that records per-layer intensity values for each bundle. We extended the analysis code to offer three alternative aggregation modes for per-bundle fluorescence intensity (shown in Fig 10): (i) the sum of raw intensities across all detected layers for each bundle; (ii) the sum restricted to the top (n) layers, where (n) is the largest depth shared by all bundles being compared; and (iii) the sum obtained by padding shallower bundles to (n) layers by repeating the bundle’s weakest-layer value, where n is the maximum depth across bundles.**”

The new submitted figure is below:

**9) For figure 11, I am not convinced as to how useful this tool will be for other datasets from other ages. It seems like you would have to retrain it using your own images, but if you have already marked the tonotopic position of each image beforehand than what value does this feature provide?**

**tl;dr:** We agree the current model is a proof-of-concept and have clarified this in the text. Its purpose is to demonstrate feasibility and encourage community data sharing to build a more generalizable foundation model.

**Location of Changes:** The text in Results (Section 1.4.5) and Discussion (Section 2, page 20) has been revised to frame this feature as a proof-of-principle and a goal for future collaborative work.

We appreciate the reviewer’s comment and fully agree that, in its current state, the tonotopic region prediction feature may have limited direct utility for other labs. As described, the current model was trained specifically on data from postnatal day 5 (P5), and its predictions are therefore most reliable for that age group.

However, we view this feature as a proof of principle that demonstrates the feasibility of predicting anatomical region (i.e., tonotopic position) directly from volumetric image stacks without relying on manual labeling or prior knowledge. This is an important technical milestone. It opens the door to future development of generalizable tonotopic prediction models that could work across a range of ages and species, provided that sufficient training data becomes available.

We believe this approach can be especially useful in large-scale or high-throughput studies where precise metadata annotation is unavailable or impractical. A reliable automated prediction of a region could assist in data organization, facilitate comparative analyses, and enable downstream modeling without the need for manual pre-sorting or human input.

Just as importantly, we believe that such classification models can eventually be used to extract new knowledge about the key morphological features that change with tonotopic position, hopefully leading to new fundamental understanding of the underlying biology of the cochlea. Similarly, while automated analyses depend heavily on accuracy and robustness, it must also be pointed out that if/when a model fails in a different biological context, this can lead to new biological discoveries by querying why/where/when the model failed.

By publishing this preliminary model, we hope to encourage the community to build upon this work, especially by contributing regionally annotated datasets from various ages and species. With collaborative efforts, this could eventually lead to the creation of a foundation model capable of robust region prediction and explanation across diverse biological conditions, which would significantly benefit cochlear research and related fields.

**10) For figure 12 and 13, could you compare your measurements to those collected using a different method or program?**

**tl;dr:** We have now validated our automated angle measurements against manual two-point measurements in Fiji for both datasets and found no statistically significant difference, confirming our method's accuracy.

**Location of Changes:** New statistical comparisons have been added to Results (Section 1.4.6, page 14). Supplementary Tables S5 and S6 contain the full per-bundle measurement data for this validation.

**Important: Figure 12 and 13 are now 13 and 14**

**Answer:** Thanks for reviewer comment and we agree that we have not included any comparison or any statistics analysis for angle measurements. We asked our biologist team to manually measure the angles using Fiji. Our team manually selected two points per bundle and computed the orientation angle using the same arctangent formula applied in VASCilia, ensuring a direct and fair comparison.

**All (x, y) coordinate measurements used for angle computation and collected in Fiji are provided as CSV files in the supplementary materials accompanying the paper.**

**We’ve added this paragraph to the paper in section 1.4.6**

**For Figure 13, the Fiji measurements had a mean of 94.04°, median of 92.32°, and standard deviation of 8.83°, while VASCilia reported a mean of 93.36°, median of 92.47°, and standard deviation of 8.44°. The Wilcoxon signed-rank test gave a p-value of 0.0652, and the paired t-test yielded a p-value of 0.0834, indicating no statistically significant difference. Similarly, for Figure 14, Fiji results showed a mean of 87.94°, median of 87.73°, and standard deviation of 7.80°, while VASCilia produced a mean of 86.88°, median of 85.75°, and standard deviation of 5.90°. The Wilcoxon test returned a p-value of 0.1901, and the paired t-test gave a p-value of 0.2738, again confirming no statistically significant difference. These results validate that VASCilia produces automated angle measurements that are consistent with manual Fiji annotations. See Tables S5 and S6 in the supplementary materials that record all comparison values.**

**Reviewer #2** **(identities themselves as Bo Zhao): The study "VASCilia (Vision Analysis StereoCilia): A Napari Plugin for Deep Learning-Based 3D Analysis of Cochlear Hair Cell Stereocilia Bundles" by Kassim and colleagues developed a fantastic deep-learning based software capable of automatically and efficiently measuring many key cellular properties of hair cells including stereocilia length, actin content, and cell polarity. These features are critical for maintaining normal hearing function, and defects of them are known to contribute to hearing loss. Compared with manual measurements using tools such as Fiji, which are often time-consuming and labor-intensive, this automated approach offers a significant advancement. The reviewer greatly appreciates the valuable tools the team has brought to the field. To further strengthen this manuscript and potentially enhance the software's performance, I have the following suggestions.
 1. The sentence in the abstract stating that "Using VASCilia, we found that the total actin content of stereocilia bundles (as measured by phalloidin staining) does not necessarily increase with bundle height, which is likely due to differences in stereocilia thickness and number" could be clarified further, as it currently lacks essential information such as the genotypes and developmental stages of the hair cells being referenced.**

**tl;dr:** We agree and have revised the abstract to specify the finding applies to 5-day old normal and Eps8 KO mice. We also created a dedicated case study section in the Results to consolidate this analysis.

**Location of Changes:** The Abstract has been edited to include the genotypes and age. A new Results Section 1.4.3 ("Case Study 1...") has been created to centralize this finding.

**Answer:** We appreciate the reviewer’s comment and agree that the abstract statement could be clearer regarding the experimental context, including genotypes and developmental stages. To address this, we have updated the abstract to include explicit reference to the **genotypes (WT vs KO) and the developmental stage (P5)**. We added to the abstract ‘(**as measured by phalloidin staining in both normal and *Eps8* KO 5-day old mice}**) ‘, Additionally, we recognized that the relevant observation and analysis were previously distributed across two sections, potentially limiting clarity for readers. **To improve this, we have reorganized the manuscript by introducing a dedicated subsection titled**

**"1.4.3 Case Study 1: Tonotopic Patterns of Bundle Height and Phalloidin Signal (WT vs Eps8 KO)" immediately following the Validation of Stereocilia Bundle Height Measurement and Fluorescence Intensity Measurements subsections. This new subsection consolidates our analysis, results, and interpretations related to the correlation between bundle height and actin intensity.**

This reorganization ensures that readers can easily locate the supporting data and contextual details referenced in the abstract. It also enhances the logical flow of the Results section by presenting the validation of height measurements, intensity quantifications, and their biological interpretations in a sequential and cohesive manner. We believe these revisions clarify our findings and better support the claims made in the abstract.

**The code and data for generating this case study is available in the new** released version **VASCilia 1.4.0**

[**Napari-VASCilia/downstream_analysis/WTvsKO_casestudy.py at main · ucsdmanorlab/Napari-VASCilia**](https://github.com/ucsdmanorlab/Napari-VASCilia/blob/main/downstream_analysis/WTvsKO_casestudy.py)

**2. The fluorescence intensity measurements in Fig. 10 appear to show edge effects. Specifically, the intensity of OHCs in the upper left corner is much lower compared with those OHCs on the right side.**

**tl;dr:** We investigated and found no spatial edge effect; the perceived difference was due to variations in bundle segmentation depth. We have updated Figure 10 and our analysis options to control for depth, which resolves the apparent discrepancy.

**Location of Changes:** This response is the same as for Reviewer #1, Comment 8. Figure 10 was fully revised, and a clarifying paragraph was added to the Discussion (Section 2, page 18).

We sincerely thank the reviewer for this insightful observation. The observed discrepancy is largely related to the depth (z-extent) of the segmented bundles. Specifically, during imaging, certain bundles may appear shallower along the z-axis due to factors such as reduced phalloidin staining, partial photobleaching, signal attenuation with imaging depth, or limitations in optical sectioning and resolution. Additionally, biological variability in stereocilia bundle density and labeling can also affect the depth captured during segmentation.

Since our total intensity measurements are computed based on the full 3D segmented volume, bundles with fewer segmented layers may exhibit lower total intensities, even when their per-layer intensity is comparable to others. Your comment inspires us to generate an additional csv file, called **All_bundles_per_layer_intensity.csv**

Through the Per-Layer Intensity Profiling feature, biologists are now equipped with flexible modes to manage depth variability in intensity measurements. This is how the All_bundles_per_layer_intensity.csv file appears for the first five cells. In this example, all cells have the same depth, but variable depth is also possible.

We revised the bundle‐intensity plots to show **raw, pre–background-subtraction** phalloidin values (instead of background-subtracted). We also changed the normalization to **max-scaling** (value ÷ per-plot maximum) rather than min–max, which avoids forcing any bundle to zero (such as bundle 38) while preserving relative differences. We also added code to generate three bar-plot modes: **(1) total raw intensity across all layers** **(2) total raw intensity for the top *n* layers**, where *n* is the largest count shared by all bundles; and **(3)** **total raw intensity after padding missing layers** by repeating each bundle’s weakest layer value. These options let biologists choose the most appropriate view.

To further illustrate this, we have extended Figure 10 to include additional views of four example cells—two inner hair cells (IHCs) and two outer hair cells (OHCs). For each selected cell, we present six consecutive 2D z-slices along with the final 3D view of the segmented bundle. This expanded view provides a more detailed depiction of the segmentation boundaries and how they relate to the underlying actin signal across depth.

**For this reason, we introduce a new paragraph in the Discussion section:**
 “**One potential limitation when measuring total intensity between bundles is the variability in segmentation depth across cells, which can arise from differences in fluorescence signal strength (phalloidin here), imaging depth, or biological heterogeneity. This variability may cause some bundles to appear shallower, potentially affecting their total intensity measurements. Although such differences tend to average out when large numbers of cells are analyzed, minimizing their impact on overall trends and group comparisons, we also provide a dedicated option for researchers who wish to control for this factor. Specifically, VASCilia outputs a CSV file that records per-layer intensity values for each bundle. We extended the analysis code to offer three alternative aggregation modes for per-bundle fluorescence intensity (shown in Fig 10): (i) the sum of raw intensities across all detected layers for each bundle; (ii) the sum restricted to the top (n) layers, where (n) is the largest depth shared by all bundles being compared; and (iii) the sum obtained by padding shallower bundles to (n) layers by repeating the bundle’s weakest-layer value, where n is the maximum depth across bundles.**”

**3. Would it be feasible to measure the height of the lower rows of stereocilia and quantify the height differences between different rows? Since different rows of stereocilia frequently overlap at the basal region, is there a way to identify the base of each stereocilium based on 3D images, such as through counterstaining with a stereocilia basal marker?**

**tl;dr:** This is an excellent future direction, but it is currently beyond the scope of our tool as it would require stereocilium-level segmentation and higher-resolution imaging data. We have clarified this limitation in the manuscript.

**Location of Changes:** This is now addressed in the new "Limitations and Future work" paragraph in the Discussion (Section 2, page 20).

We thank the reviewer for this insightful suggestion. Measuring the lower rows of stereocilia is indeed important but remains challenging due to the current imaging resolution.

Our future direction, which we are actively pursuing, is to develop methods to identify the three stereocilia rows and segment individual stereocilia. This will expand the framework’s capability to address more complex structural questions. The framework is adaptable and can be extended to incorporate additional imaging channels for basal markers, provided that staining quality and imaging resolution are sufficient; efforts our hearing research team is currently advancing. We plan to explore this direction further in collaboration with them to improve the resolution and quantification of stereocilia bundle architecture across different rows. This will likely require STED, expansion, or correlative light-electron microscopy to accomplish. Our hope is this work will encourage more data sharing from other labs that have such data on hand already.

**Several minors:
 1. Please consider labeling all the three OHCs in Fig. 1 using the same color.**

I'm not sure if you're referring to Fig. 1 or Fig. 10. I believe you mean Fig. 10. If that's the case, this issue has already been addressed. Additionally, the figure has been expanded to include more cell examples, displaying their layers in 2D along with the corresponding intensity profiles.

**2. There is a typo in the last sentence of page 3.**

Thanks for your observation, the typo has been fixed

**3. The unit of length is missing in the Y-axis in Fig. 9a.**

Sure we added it to the figure
 **4. To improve clarity, please specify that it is the length of the tallest row of stereocilia in Fig. 9a.**
 We’ve added to the legend ‘The unit of length for measuring bundle height is in microns (μm), defined here as the length of the tallest row of stereocilia within each bundle.’

**Reviewer #3:** **This manuscript presented a very useful tool for studying the morphology of auditory hair cell stereocilia. I tried this plugin and the overall quality of it is solid, with tutorials and functions well documented. It has potential to be adopted by other labs to streamline hair bundle analysis. That said, there are still some questions about VASCillia that could be addressed for improving its clarity and utility. Please check the comments below:**

**1) Versatility of VASCillia: Considering that vestibular hair cells are widely used for studying PCP phenotypes, can VASCillia be easily adopted to study those hair cells with retraining the model?**

**tl;dr:** While the current model is trained on cochlear cells, we designed VASCilia with a built-in training module that allows users to easily fine-tune the model for other cell types, like vestibular hair cells, using their own annotated data.

**Location of Changes:** This is clarified in the "Limitations and Future work" paragraph in the Discussion (Section 2, page 20).

The current VASCillia model is specifically trained on cochlear hair cells. However, we designed the training module within the plugin precisely to address such needs. Users can efficiently generate their own ground truth data through an integrated, fast, and user-friendly interface. This enables them to fine-tune our existing model on their specific datasets, such as vestibular hair cells, directly within the same plugin. Here is, for example, a processed versatile image dataset that has been annotated using our Training section. Once the user finishes processing a considerable number of cells (up to 500 cells), they can press ‘Train New Model for 3DBundleSeg’. Just to confirm, the image below is just a manual annotation example that we did quickly to show you how the user can annotate through our plugin. It is not a dataset that we used to train vascilia.

We hope to update the GitHub with such a model after we are able to collect sufficient image data and complete the manual annotation.

The details of how to use the training section are available in the paper in Figure 45, 46, 47, 48, 49, and 50. Also they are available here [Commuting Training Action — VASCilia 1.3.0 documentation](https://ucsdmanorlab.github.io/Napari-VASCilia/features/train_section.html)

**2) 3D contour of hair bundles: From the representative images provided by Figure 6, most of the reconstructed 3D contours seem to have a "plateau" at the top of the hair bundle, which may not accurately represent the true staircase-like structure of a hair bundle. I wonder if this could be improved by retraining the model. Or whether the tip regions are excluded by design that they are classified into pre-cellular zone.**

**tl;dr:** This plateau appearance is a limitation of the original imaging resolution, not the model itself. The model can be retrained on higher-resolution data to capture finer structures, and we encourage data sharing to enable this.

**Location of Changes:** This point is also addressed in the "Limitations and Future work" paragraph in the Discussion (Section 2, page 20).

The appearance of the plateau in the reconstructed 3D contours is mainly due to the limited resolution of our training data, which does not capture the intrinsic details of the true staircase-like structure of hair bundles. Such fine structural features are typically more apparent in electron microscopy images, which offer much higher resolution compared to standard light microscopy.

If higher-resolution segmentation of the staircase-like architecture is desired, researchers can use the plugin's training module to re-train or fine-tune the algorithm with their own higher-resolution datasets. However, we would like to emphasize that manual annotation is resource-intensive, and our lab does not have the capacity to provide annotations that address every imaging modality or biological question. Our hope is that this paper encourages more data sharing from labs that have higher resolution data (e.g. STED or expansion or even correlative light-electron microscopy datasets on hand)!

That said, we have made every effort to deliver the first robust 3D model capable of handling cochlear microscopy images across different species and developmental stages, and we have designed the plugin to be adaptable for further refinement by the community

**3) Stereocilia length quantification: The automatic quantification of the stereocilia length is very powerful. However, it is important to note that VASCillia only quantifies the longest stereocilia within each bundle. Since VASCilia identifies the 3D contour of the hair bundle, I am curious if its function can be extended to infer the height of the second row of stereocilia by using the contour size of each z-section. This function could be very helpful to study the actin remodeling after noise damage or MET deficit. But I understand this could be very challenging to implement.**

**tl;dr:** This is the same valuable suggestion as Reviewer #2's. It would require stereocilium-level segmentation and higher-resolution data, which is an important goal for future work.

**Location of Changes:** This is addressed in the "Limitations and Future work" paragraph in the Discussion (Section 2, page 20).

**Long answer:** We acknowledge that our height quantification is limited to the highest stereocilia within each bundle and does not provide measurements for the height of individual stereocilia or distinct bundle rows. Many of the current limitations of VASCilia stem from the resolution constraints of our imaging data, which is insufficient for capturing the finer structural details needed for precise quantification. Additionally, our current model is not designed for stereocilium-level segmentation, which would require much higher-resolution datasets, such as those obtained from STED or expansion microscopy. Developing such a specialized model is beyond the scope of this work and represents a direction for future studies. While manual inspection might allow experts to identify individual stereocilia in select cases, our automated analysis system is not designed for that level of granularity.

**4) Validation of the stereocilia length quantification: It would be helpful to include human annotator ground truth of Esp8 KO mice in figure 9a. Also, I wonder if VASCilia can also provide accurate quantification for hair bundles with severe developmental deficit, such as MYO7A or CDH23 KO hair cells.**

**tl;dr:** An excellent suggestion. We have now validated VASCilia's measurements against manual Fiji measurements on both Eps8 KO and severely disorganized Cdh23-/- bundles, finding no significant difference. We further expanded this into a major new case study demonstrating that VASCilia can quantify complex textural disorganization.

**Location of Changes:** Validation on KO models was added to Results (Section 1.4.1, page 8), referencing new Supplementary Tables S7 and S8. The new Results Section 1.4.4 ("Case Study 2...") and the corresponding Figure 11 showcase this new capability. The Abstract was also updated to include this major finding.

**Answer for first sentence:** We appreciate the reviewer’s suggestion. To validate our automated stereocilia length quantification we manually annotated stereocilia lengths in six **Eps8 KO datasets**—two from the base, two from the middle, and two from the apex regions. From all regions, 15 cells were randomly selected. Using Fiji, the biologists identified the tallest stereocilium in each cell and measured its length from the base (as determined by scrolling through the Z-stack) to the tip.

We compared these manual measurements against the results from our automated VASCilia quantification pipeline. The comparison showed no statistically significant difference between the two methods. Specifically, the mean stereocilia length was 1.8258 µm for Fiji and 1.8353 µm for VASCilia, with standard deviations of 0.4263 µm and 0.4045 µm, respectively. A Wilcoxon signed-rank test and a paired t-test both confirmed the absence of significant differences.

**Answer for second sentence:** To answer this question, we used data from ***Cdh23* KO mice**. We first segmented the bundles using VASCilia. Although the algorithm was not trained on such data, it successfully segmented the structures. Based on the segmentation results, we measured the bundle heights of all cells in one sample (15 cells) using both VASCilia and Fiji. We found no significant difference in bundle height between the two methods, as shown in the table below:

**We added this paragraph to the paper in section 1.4.1 and we also uploaded the complete set of paired observations as a CSV file that has all measurements details used in Fiji:**

**To further validate stereocilia–length quantification, we compared VASCilia against manual Fiji measurements on n=15 *Eps8-KO* cells sampled from six stacks (two Base, two Middle, two Apex) drawn from the dataset used in Fig. 9a. We also evaluated n=15 cells from animals lacking functional *Cdh23* (*Cdh23^v-6J/v-6J^*, referred to as *Cdh23^-/-^*) —chosen to represent severely dysmorphic bundles and not included in the main case study—to assess generalization. Across both cohorts, the mean lengths were highly comparable and the paired differences were not statistically significant (paired t-test and Wilcoxon signed-rank); see Tables S7–S8.**

**More Experiments for *Cdh23* cells unrelated but inspired by your comment**

Having this dataset from our collaborator inspired us to include an additional case demonstrating how VASCilia can be used to quantify other measurements beyond bundle height and intensity measurements (as we have not included such a case). Once the bundles are segmented, VASCilia enables the extraction of various metrics that can support the conclusions of many studies. For example, we analyzed two datasets: one from heterozygous mice (normal controls), referred to as "***Cdh23* + /−**," and another from ***cdh23*** knockout mice, referred to as "***Cdh23* − /−**." The ***Cdh23* + /−** dataset contains well-organized bundles, while the ***Cdh23* − /−** dataset displays disorganized bundles. In total, we analyzed 187 crops from ***Cdh23* + /−** cells and 176 crops from***Cdh23* − /−** cells.

To quantitatively assess this difference, we extracted texture-based features from segmented bundle image crops using VASCilia. Specifically, we computed Gray Level Co-occurrence Matrix (GLCM) properties—contrast, correlation, energy, and homogeneity—as well as Local Binary Pattern (LBP) histograms for each crop. Statistical comparison using the Mann-Whitney U test revealed highly significant differences between the two genotypes across all GLCM features (p < 0.0001).

For example, ***Cdh23* − /−** samples exhibited **higher median** contrast (136.88 vs. 74.76), which makes sense given the more disorganized and heterogeneous texture of the bundles, leading to greater intensity variation between neighboring pixels. **Correlation was lower** in ***Cdh23* − /−** samples (0.98 vs. 0.99), reflecting weaker spatial relationships and less structural regularity in disorganized bundles. **Energy**, which measures textural uniformity, was also **lower** (0.0147 vs. 0.0157), indicating a more complex, less repetitive texture pattern. Finally, homogeneity was reduced in ***Cdh23* − /−** samples (0.1368 vs. 0.1782), consistent with greater local contrast and uneven intensity distribution in their disorganized structures. Significant differences were also observed in several LBP histogram bins, including bin 0, bin 1, bin 2, bin 5, bin 6, bin 7, and bin 9 (p < 0.01). These bins correspond to common local binary patterns representing uniform or near-uniform texture regions. The shifts in their distributions likely reflect changes in local texture caused by the disorganized morphology in ***Cdh23* − /−** bundles compared to the more uniform structure in ***Cdh23* + /−** samples.
 Dimensionality reduction techniques (PCA and t-SNE) visually revealed a clear separation between the two genotypes in feature space. To quantitatively validate this observation, we trained a Random Forest classifier on the extracted features. The model achieved 94.5% accuracy in distinguishing between ***Cdh23* + /−** and ***Cdh23* - /−** samples, with only four misclassifications out of 73 test instances. The most important features identified by the classifier aligned with those that were statistically significant.

Here is also a representative image for ***Cdh23^+ /-^*** and ***Cdh23* ^- /-^** samples with corresponding segmentation

**This case study has been added as a new study in the paper, see section 1.4.4. All extracted features have been uploaded. The code and data for generating this case study is available in the new** released version **VASCilia 1.4.0** [**Napari-VASCilia/downstream_analysis/homo_vs_het_casestudy.py at main · ucsdmanorlab/Napari-VASCilia**](https://github.com/ucsdmanorlab/Napari-VASCilia/blob/main/downstream_analysis/homo_vs_het_casestudy.py)

**Case study 2: Quantifying bundle disorganization (*Cdh23^+ /-^* vs *Cdh23^- /-^*)**

**We present an illustrative case study demonstrating VASCilia’s utility beyond bundle-height and intensity measurements. We analyzed 3D image stacks from *Cdh23^v-6J/v-6J^* mice, referred here as *Cdh23^- /-^* (Indzhykulian et al., 2013) to quantify texture differences between genotypes. We compared heterozygous controls *Cdh23^+ /-^* ; n=187 crops) with homozygous knockouts (*Cdh23^- /-^*; n=176 crops). *Cdh23^+ /-^* bundles were well organized, whereas *Cdh23^- /-^* bundles were visibly disorganized, consistent with the expected phenotype.**

**To quantify these differences, we extracted texture features from segmented bundle crops using VASCilia. Specifically, we computed gray-level co-occurrence matrix (GLCM) features—contrast, correlation, energy, and homogeneity—and Local Binary Pattern (LBP) histograms (Pietikäinen et al., 2011) for each crop. *Cdh23^- /-^* samples had higher median contrast (136.88 vs 74.76), consistent with more heterogeneous texture; lower correlation (0.98 vs 0.99), reflecting weaker spatial regularity; lower energy (0.0147 vs 0.0157), indicating less uniformity; and lower homogeneity (0.1368 vs 0.1782), consistent with greater local contrast. Two-sided Mann–Whitney U tests showed highly significant differences across all GLCM features (p<0.0001). Several LBP bins (0, 1, 2, 5, 6, 7, 9) also differed between genotypes (p<0.01), reflecting shifts in local texture patterns. Dimensionality reduction (PCA and t-SNE) visually revealed a clear separation between genotypes in feature space. To quantitatively validate this observation, we trained a random forest on the extracted features (training n=290; test n=73), which achieved 94.5% accuracy on held-out crops (4/73 misclassified). The most important features aligned with those that were statistically significant (see revised Figure 11; representative examples in Supplementary Figure S5).**

**Notably, by using VASCilia, we were able to complete this entire analysis of this new case in just a few hours, an effort that would typically take a biologist several weeks or even months to achieve manually.**

**5) Fluorescence Intensity Measurements: Could the authors elaborate on the method used to normalize fluorescent signal intensity? For example, what are phalloidin intensities normalized to in figure 9b and 10? Are intensities calculated as total signal, or volume-normalized as signal/voxel? More detail on the normalization strategy would be helpful to minimize batch effects and replicate these analyses.**

**tl;dr:** We have clarified the normalization methods for both figures. Figure 9b uses global min-max normalization for comparability, while Figure 10 uses per-stack max normalization for visualization. We also now offer three depth-controlled analysis modes.

**Location of Changes:** The normalization method for Figure 9b is now described in its corresponding Results section (1.4.3). The methods for Figure 10 are detailed in the Discussion (Section 2, page 18) and the figure caption.

**For Figure 10**, this is provided as an example of the plots automatically generated and stored in the Intensity_responce folder when a user processes a dataset with VASCilia. The intensities shown in the old figure (submitted version) are min-max normalized within the current sample only for visualization purposes. The original, unnormalized intensity values are preserved in two accompanying CSV files: one containing the total and mean intensities per bundle in 3D **(region_intensities.csv)**, and another containing the total intensity per Z-layer **(All_bundles_per_layer_intensity.csv)**, enabling users to investigate intensity distributions across layers if they have concerns about any total intensity measurement.

**We re-generated Figure 10 using max normalization (scaling each bundle by its own maximum) rather than min–max normalization to avoid forcing any bundle to zero in the plot (such as bundle 38), and we removed the background-subtraction step to show the raw values. The analysis code now supports three modes: mode 1: sums intensity across all available layers for each bundle; mode 2: sums only the top *n* layers, where *n* is the largest depth shared by all bundles; and mode 3: pads shallower bundles to *n* layers by repeating each bundle’s weakest-layer value before summing. The corresponding plots and CSV outputs for modes 1, 2 and 3 are provided here and in the Supplementary Materials.**

We have also extended Figure 10 to display four example bundles **(Cells 3, 7, 26, and 18)**, showing the raw intensity at each Z-layer and the corresponding total intensity in 3D. This demonstrates that differences in total intensity, when depth is constant, are primarily driven by how large and dense each bundle is, providing clearer insights into the biological variability of fluorescence signals.

Moreover, users can compute not only phalloidin intensities, but also any other fluorescence channel, such as Eps8, MyoXVa, or others, by specifying the desired channel in the configuration file. These steps and customization options are detailed in the VASCilia documentation.
 [Signal Computation — VASCilia 1.3.0 documentation](https://ucsdmanorlab.github.io/Napari-VASCilia/features/Compute_fluorescense_intensity.html)

**In Figures 9b**, fluorescence intensities were computed as the total intensity per stereocilia bundle (**3D sum within each bundle)**, extracted from the **region_intensities.csv** file, specifically the 'Total Intensity' column.

To account for batch effects and variability across samples, we performed global min-max normalization across all samples (WT and KO combined), the normalized intensity was calculated as:

Global Min/Max were computed on the combined dataset across all conditions (WT and KO).

This ensures a 0–1 scaling across all data points, making intensities comparable across experimental conditions and batches.

The intensities are not volume-normalized (i.e., not divided by voxel count) but represent the sum of voxel intensities within each bundle segmentation.

This normalization approach ensures that:

- Differences in intensity reflect biological differences rather than batch or imaging variability.
- It enables direct comparison of intensities across WT and KO samples, tonotopic regions, and cell classes (IHCs vs OHCs).

We re-generated Figure 9b using the code option (mode2) that restricts summed intensity to the top *n* layers (where *n* is the largest depth shared by all bundles being compared). Under this setting, the Apex→Base tonotopic gradient is more pronounced. For more details about statistical differences, see our response to Reviewer 1, Comment 7.

**New Figure using Mode2:**

**For this reason, we’ve added this paragraph to section 1.4.3 in the paper to illustrate Figure 9b normalization step.
 ‘Intensities are computed as the 3D sum within each bundle and global Min-Max normalized across all WT+KO samples to place values on a scale of 0 to 1. In this analysis, we applied mode 2, discussed in Section 2, separately to each dataset. For a given data set D, we set n to the largest depth shared by all bundles within D; equivalently, n= min{bundle depths in D}. This dataset-specific choice of n ensures that within each data set, all bundles contribute the same number of layers.’**

**We also updated section 1.4.2 (which describes Figure10) by mentioning that the intensities in the plot are max normalized**

**The code and data for generating this case study is available in the new** released version **VASCilia 1.4.0**

[**Napari-VASCilia/downstream_analysis/WTvsKO_casestudy.py at main · ucsdmanorlab/Napari-VASCilia**](https://github.com/ucsdmanorlab/Napari-VASCilia/blob/main/downstream_analysis/WTvsKO_casestudy.py)

**6) High-Throughput Computation of Bundle Orientation: It is a very useful function of quantifying the angle of stereocilia. I wonder if the author could provide some validation of cochlea with PCP deficit. Meanwhile, is it easy to quantify the V-shape angle of a hair bundle?**

**tl;dr:** We have now validated the orientation tool on an independent dataset of PCP-deficient (*Pcdh15-R245X*) mouse cochleae, showing strong agreement with manual Fiji measurements and confirming its utility for disorganized phenotypes**.**

**Location of Changes:** This validation is now included in Results (Section 1.4.6, page 15). Supplementary Table S9 and Supplementary Figure S4 contain the data and visualization for this new validation.

We thank the reviewer for this suggestion**.** In response, we analyzed an independent cochlear dataset with planar cell polarity (PCP) deficits provided by collaborators [2]. Early postnatal Pcdh15-R245X mouse cochleae were acutely dissected, fixed with 4% PFA, stained with phalloidin to visualize stereocilia bundles, then mounted and imaged with Leica SP8 confocal microscopy using a 63x 1.3NA lens.

VASCilia successfully segmented and measured the orientation angles of all bundles.

To validate our method, we used Fiji following the same two-point strategy and computed the angle using arctan for all the bundles (46 bundles). The results confirmed that our approach is accurate and valid, with no statistically significant difference:

We also note that computing the angles manually in Fiji takes approximately 5 hours for those 46 bundles, whereas in VASCilia, the measurements are generated instantly. If any adjustments are needed, the user can make them in under 5 minutes for the entire dataset.

The angle tool is a semi-supervised method that offers the flexibility to perform high-throughput computation while allowing easy correction in challenging cases—making it robust and adaptable. You can see here that all the pink points are editable and adjustable.

**All Fiji measurements have been stored in a CSV file and are uploaded to the supplementary materials. We’ve added this text to section 1.4.6.**

**We also quantified bundle orientation angles in PCP- deficit cochleae to assess generalization. The early postnatal Pcdh15-R245X mouse cochleae were acutely dissected, fixed with 4\% PFA, stained with phalloidin to visualize the stereocilia bundles, then mounted and imaged with Leica SP8 confocal microscopy using a 63x1.3NA lens. Validation against manual Fiji measurements showed comparable means with no statistically significant difference (Table S9; Fig S4 in the supplementary materials.) }**

**Lastly, to answer ‘Meanwhile, is it easy to quantify the V-shape angle of hair bundle?’**
 VASCilia does not currently quantify the internal V-shape angle of the hair bundle, as its measurements are based on two points along the bundle to determine overall orientation. This would be a great application for future development, ideally as part of a stereocilium-segmentation model.

**7) Other comments: There are some missing definitions for the abbreviations, such as "FC" in Figure 2 convolutional neural network. Please include that in the manuscript for improving readability.**

**Answer:**

Thank you for bringing this to our attention. We have updated Figure 2 caption to include all missing definitions to improve readability. Specifically, we have added the following clarification to the caption:

**“For ZFT-Net, Conv represents CONVolution layers, BN stands for Batch Normalization, ReLU is the Rectified Linear Unit activation function, Pool refers to Pooling layers, and FC denotes Fully Connected layers.”**

This addition ensures that all abbreviations in the figure are clearly defined for the reader.

**New References added to the paper and cited here:**

1. Indzhykulian AA, Stepanyan R, Nelina A, Spinelli KJ, Ahmed ZM, Belyantseva IA, Friedman TB, Barr-Gillespie PG, Frolenkov GI. Molecular remodeling of tip links underlies mechanosensory regeneration in auditory hair cells. PLoS Biol. 2013;11(6):e1001583. doi: 10.1371/journal.pbio.1001583. Epub 2013 Jun 11. PMID: 23776407; PMCID: PMC3679001.
2. Ivanchenko MV, Hathaway DM, Klein AJ, Pan B, Strelkova O, De-la-Torre P, Wu X, Peters CW, Mulhall EM, Booth KT, Goldstein C, Brower J, Sotomayor M, Indzhykulian AA, Corey DP. Mini-PCDH15 gene therapy rescues hearing in a mouse model of Usher syndrome type 1F. Nat Commun. 2023 Apr 26;14(1):2400. doi: 10.1038/s41467-023-38038-y. PMID: 37100771; PMCID: PMC10133396.
