# Supplementary material for: VASCilia is an open-source, deep learning-based tool for 3D analysis of cochlear hair cell stereocilia bundles
Source: PLoS Biol. 2026 Jan 20;24(1):e3003591. doi: 10.1371/journal.pbio.3003591 (PMC12829968; doi:10.1371/journal.pbio.3003591)
Supplement: Attachment — Submitted filename: PLOS Biology response to reviewers.docx [file pbio.3003591.s024.docx]

Reviewer #1: The authors have made some significant improvements to the manuscript in response to the reviewers' comments, although the length of the responses made the document difficult to read. We do appreciate that the authors provided a summary of the responses and then followed that with more details. Nevertheless, the manuscript is not an easy read.

Overall, we think the paper presents what could be a useful tool for some hair cell biologists and does a decent job of demonstrating its fidelity by comparing to other measurement techniques. Some of the specific use cases, like measuring hair bundle angles, should be very useful as the VASCilia approach allows analysis of many samples very quickly, allowing much later datasets that should have higher statistical power.

That said, this paper is in essence a methods paper and does not provide any new biological information, but rather uses case studies on some selected data sets to demonstrate each feature of the VASCilia software. As discussed below, the one case study where they claim a new biological result (reported in Fig. 9b) lacks validation and thus is difficult to accept. It is not also clear whether this tool will prove more useful than commercially available solutions (which admittedly will also require a lot of work to train and configure).

We appreciate the suggestion. We have added a dedicated paragraph in the Discussion section of the manuscript comparing VASCilia to commercial tools like Imaris and Arivis. We highlight that while commercial tools are powerful, VASCilia offers an open-source, reproducible, and domain-specific alternative that eliminates the "black box" nature of proprietary deep learning modules.

Thus this manuscript is an interesting presentation of a novel tool that may be useful to a subset of hair cell biologists. The tool is unlikely to be the only one used by those in the field, however, and there remain lingering questions about how easy it is to use for anyone trying to set it up in their laboratory.

Concerns and comments

1. A concern from the original review was over challenges in installing and using the program due to insufficient installation instructions and description of system requirements. The authors addressed these concerns and their new installation guide that includes system requirements seems much more thorough and easier to follow. Moreover, the text is certainly more streamlined now due to removing the user guides. Nevertheless, we were unable to get the program running ourselves because it apparently does not recognize the GPU we have in the computer. We recognize that in order to properly test the program we need to match the specifications laid out precisely, but it certainly limits the utility of the program if a new dedicated system must be purchased.

*Tl;dr: We apologize that the previous version did not run on your specific hardware. To address this, we have conducted rigorous testing across a wide range of consumer and workstation GPUs to ensure stability. We successfully validated the software on:*

- *NVIDIA RTX 3080 (10 GB VRAM)*
- *NVIDIA RTX 2080 Ti (11 GB VRAM)*
- *NVIDIA RTX 2070 (8 GB VRAM)*
- *NVIDIA RTX 4070 Ti, 4080, and 4090*

*Based on this, we have updated the documentation to explicitly state the 8 GB VRAM requirement. Furthermore, we identified that most installation friction stems from WSL (Windows Subsystem for Linux) configuration rather than the plugin itself. To resolve this, we have:*

1. *Updated the installation guide with "Troubleshooting" details related to WSL installation.*
2. *Created a step-by-step video tutorial (linked in the repo) demonstrating the full setup process.*

*We are confident these measures ensure accessibility for the field.*

Since the specific error or issue encountered by the reviewer was not described, it was difficult for us to reproduce and resolve the problem directly. We have carefully reviewed our GitHub repository and confirmed that no new issues or installation errors have been reported since the last revision.

To ensure that this concern is fully addressed for both the reviewer and the editorial board, we asked biologists from different labs (with no prior experience installing software packages), to install the program; altogether they installed VASCilia on three different computers with different specifications and the package ran successfully on all of them:

They ran it successfully on:

NVIDIA RTX 3080 (10 GB VRAM)
NVIDIA RTX 2080 Ti (11 GB VRAM)
NVIDIA RTX 2070 (8 GB VRAM) — verified with screenshots showing successful execution

Also we tested the software in more computers in our own laboratory including:

NVIDIA RTX 4070 Ti (12 GB VRAM)

NVIDIA RTX 4080 (16 GB VRAM)

NVIDIA RTX 4090 (24 GB VRAM) — verified with screenshots showing successful execution

Based on these tests, we have updated the installation guide to indicate that **8 GB VRAM** is sufficient, and we have listed the specifications of all tested systems in the installation page. These results confirm that the package is compatible with a wide range of hardware configurations and does not require a specialized or dedicated system.

During the installation process, we noted that most of the challenges users encountered were related to **Windows Subsystem for Linux (WSL)** setup and **configuration file path settings** rather than the software itself. We have now documented all of these potential issues and their solutions in the installation guide [Installation — VASCilia 1.3.0 documentation](https://ucsdmanorlab.github.io/Napari-VASCilia/installation.html).

Furthermore, to support users with varying levels of technical experience, we have prepared a **step-by-step video tutorial** [VASCilia Installation YasminKassim](https://www.youtube.com/watch?v=9snkuhpKN3c) demonstrating the entire installation process — including WSL setup, package installation, and configuration file path settings. We believe these additions will significantly improve accessibility and help future users avoid similar issues.

2. The authors are surely aware that there are commercial software programs (Arivis, Imaris, etc.) that, while not specifically designed for hair cells, already allow for 3D segmentation of hair bundles and include the ability to create deep-learning instance-based segmentation models (e.g., Arivis). However, these programs are quite expensive and having an open source program already trained for bundle segmentation will certainly be useful to hair cell biologists for certain analysis tasks. Nevertheless, we would appreciate some discussion of alternatives.

We appreciate the suggestion. We have added a dedicated paragraph in the Discussion section of the manuscript comparing VASCilia to commercial tools like Imaris and Arivis. We highlight that while commercial tools are powerful, VASCilia offers an open-source, reproducible, and domain-specific alternative that eliminates the "black box" nature of proprietary deep learning modules.

3. In the original review, we had concerns over the depth of segmentations in 3D, the possible inclusion of cuticular plate, and possible variance in its performance on flattened versus upright bundles. The authors addressed these concerns by providing a new supplementary figure that shows the segmentation with per slice overlays and orthogonal views. This figure is very helpful at providing a more thorough representation of what the segmentations look like in 3D relative to the actin signal as well as what they look like for a wider variety of bundles (in terms of both age and orientation (flat vs upright)). The changes to Fig. 10 as well as the addition of the Cdh23 -/- bundle case study were also helpful in showing the 3D segmentation performance more thoroughly for a wider variety of bundles.

Thank you for sharing your feedback

4. For the height measurements, the authors have clarified our questions as to how the manual measurements were made, provided more details on the datasets used, and provided statistical comparisons for the results. I appreciate the new supplemental figures showing the z-stacks and the excel output for each cell as well as the new validation the KO models provided in tables S7 and S8. Overall, our concerns over the height measurements have been satisfied.

Thank you for sharing your feedback

5. The use case for height measurement, bundle disorganization, and bundle orientation are the most successful because the authors include appropriate case studies using KO lines with known changes in the relevant measurement parameters and thoroughly compare their results to other measurement techniques (for height and orientation). However, the section on intensity measurements is the least successful in this regard. The authors should have used case study for intensity measurements that could be more easily validated and interpreted; without validation of their result (basal hair cells bundles more phalloidin intensity than apical hair bundles), it is impossible to know whether the results are correct. They certainly do not seem correct; we have examined thousands of cochlear hair cells labeled with phalloidin, and we are very surprised by the authors' claimed result. The only way that data in Fig. 9b should be included is if the result is corroborated by similar

experiments with different markers (e.g., anti-actin antibody or antibody against a stereocilia cytoskeleton protein). As is, the data on total actin intensities in Fig. 9b are very hard to interpret and do not provide a good example about how these intensity measurements could be used more generally to give accurate and more high-throughput results than current measurement tools.

6. A different approach to use intensity measurements might be to use the bundle segmentations to measure the intensity of another protein expressed in hair bundles that they know would be missing or altered in expression in KO bundles (for instance, measure EPS8 expression in the WT and Eps8 KO bundles). The resulting data would be more interpretable and also easier to verify using standard measurement techniques.

We understand and appreciate the reviewer’s concern regarding Figure 10b (which was Figure 9 in the previous version). We acknowledge the reviewer’s skepticism regarding the basal-to-apical phalloidin intensity gradient in P5 mice. As suggested by the Academic Editor in the decision letter, we have not performed the additional anti-actin validation as a condition for this revision.

As noted, the data presented in this figure correspond specifically to **P5 mice**, and the observed distribution of phalloidin intensity may differ at other developmental stages. Furthermore, our measurements do not account for potential differences in the number of stereocilia in each bundle. These and potentially other variables could explain the discrepancy with the reviewer's or others’ measurements. For this reason, we added in **Figure10 caption** the following clarification:

“The tonotopic gradient observed here may not be representative of other developmental ages, which were not examined in this study.”

We would like to emphasize that our automated algorithm directly extracts the **integrated fluorescence intensity within the 3D segmented bundle**, making the measurement process straightforward and minimizing user bias or manual errors.

Finally, we appreciate the reviewer’s constructive suggestion to analyze other bundle proteins with well-characterized expression patterns. In response, we have **included new results using two additional proteins** whose differences between WT and KO lines are well established. These new examples better illustrate how *VASCilia*’s intensity-measurement module can be used for biologically interpretable and verifiable analyses.

**This analysis becomes part of Case Study 1**

**Analysis of anti-EPS8 signal in mid-cochlear inner hair cells of mature P30 WT and *Eps8* KO mice:**
 Quantitative analysis of stereocilia bundle morphology and anti-EPS8 signal in postnatal day 30 (P30) inner hair cells (IHCs) revealed pronounced structural and molecular differences between wild type (WT) and *Eps8* knockout (KO) mice. WT bundles (n = 14) exhibited a mean length of 3.59 ± 0.29 µm (SD; range: 3.12–4.28 µm), whereas *Eps8* KO bundles (n = 15) were significantly shorter, measuring 1.22 ± 0.18 µm (SD; range: 0.90–1.53 µm; Welch’s t = 25.89, df = 21.29, p = 1.4 × 10⁻¹⁷). To evaluate bundle anti-EPS8 labeling intensity, total fluorescence intensity was quantified within the same segmented IHC bundles. WT bundles exhibited a mean total intensity of 0.616 ± 0.059 (SD), while *Eps8* KO bundles showed markedly lower values of 0.094 ± 0.012. Normalization was performed by dividing each bundle’s total intensity by the maximum total intensity observed across both WT and KO groups. This reduction was highly significant (Welch’s t = 8.63, df = 14.16, p = 5.1 × 10⁻⁷). These findings demonstrate that the loss of *EPS8* leads to a substantial decrease in both the length of the stereocilia and the anti-EPS8 signal, consistent with its essential role in the promotion of F-actin polymerization and the maintenance of the stereocilia architecture.

Figure 10 has been consolidated into a single comprehensive figure that presents P5 mouse bundle height and tonotopic variations of phalloidin fluorescence intensity across all cochlear regions (*A*). In addition, the new anti-*EPS8* analysis (*panel B*) compares anti-*EPS8* fluorescence intensity in wild-type and knockout stereocilia bundles from the mid-cochlear region at P30.

**Case Study 1: Tonotopic and Maturation-Dependent Differences in Stereocilia Bundle Height and Fluorescence Signals (Phalloidin and anti-*EPS8*, WT vs. *Eps8* KO)**

**Figure Caption in the paper:**

This figure consists of two panels. Panel (A) on the left shows bundle heights and tonotopic variations of phalloidin fluorescence intensity in P5 mouse cochlear hair cells across all cochlear regions. **The tonotopic gradient observed here may not be representative of other developmental ages**, which were not examined in this study. Panel (B) presents the anti-EPS8 analysis, comparing anti-EPS8 fluorescence intensity between wild-type and knockout stereocilia bundles from the mid-cochlear region at P30.

**This analysis becomes part of Case Study 2**

***Anti-CDH23 fluorescence quantification analysis (Cdh23-/- vs. Cdh23+/-)***

To examine how loss of cadherin-23 affects anti-CDH23 staining, we quantified total fluorescence intensity in IHCs and OHCs bundles from *Cdh23*–/– and *Cdh23*+/– mice. Data were obtained from 7 *Cdh23*–/– and 6 *Cdh23*+/– datasets, all from the mid-cochlear location. Values were min–max scaled using the global all cells range across both genotypes and cell types. As shown in Fig. 11 (panel B), across all bundles, *Cdh23*–/– cells (n = 112) measured 0.202 ± 0.012 SEM, whereas *Cdh23*+/– cells (n = 76) had a mean normalized intensity of 0.482 ± 0.021 SEM. By cell type, IHC bundles were highest: *Cdh23*–/– (n = 34) 0.352 ± 0.021 SEM vs. *Cdh23*+/– (n = 24) 0.621 ± 0.040 SEM. OHC bundles showed the same trend: *Cdh23*–/– (n = 78) 0.137 ± 0.007 SEM vs. *Cdh23*+/– (n = 52) 0.417 ± 0.019 SEM.

These results demonstrate a robust, genotype-dependent reduction in anti-CDH23 bundle fluorescence in *Cdh23*–/– hair cells across both IHCs and OHCs, consistent with cadherin-23 being required to maintain normal stereocilia organization and F-actin integrity in the mid-cochlea.

Figure 12 has been reorganized into a comprehensive figure, with the texture analysis (originally presented in Figure 11 (from a to g)) displayed at the top as (A), and the newly added anti-CDH23 fluorescence quantification analysis, added to the bottom as (B).

**Anti-CDH23 fluorescence quantification analysis (Cdh23+/- vs. Cdh23-/-)**

**Figure Caption in the paper:**

This figure presents the texture analysis displayed at the top as panel (A), and the anti-CDH23 fluorescence quantification analysis included at the bottom as panel (B).

**(Panel A) [Top row]:** t-SNE of texture features [I to IV] and PCA projection show clear genotype separation in feature space, with crops from the two genotypes providing visual representation.

**(Panel A) [ I to V ]:** GLCM **energy** by genotype (*Cdh23*-/- bundles have lower energy than *Cdh23*+/-, reflecting less uniform, more complex textures; differences are statistically significant); GLCM **correlation** by genotype (*Cdh23*-/- bundles exhibit lower correlation than *Cdh23*+/-, indicating weaker spatial regularity; differences are statistically significant); GLCM **contrast** by genotype (*Cdh23*-/- bundles show higher contrast than *Cdh23*+/-, consistent with greater local intensity variation; differences are statistically significant); GLCM **homogeneity** by genotype (*Cdh23*-/- bundles show reduced homogeneity relative to *Cdh*23+/-, consistent with more uneven local texture; differences are statistically significant); and Random forest feature importance highlights key GLCM/LBP drivers.

**(Panel B):** Anti-CDH23 immunofluorescence quantification analysis for P7 IHC and OHC stereocilia bundles from a mid-cochlear location. Each data point represents a value obtained from an individual bundle, normalized to the maximum value within the dataset. Exemplar images on the right show stereocilia bundles stained with phalloidin (white) and anti-CDH23 antibody (magenta), with the automatically generated stereocilia bundle mask overlaid.
